# Deep-sea *Bacteroidetes* from the Mariana Trench specialize in hemicellulose and pectin degradation typically associated with terrestrial systems

**DOI:** 10.1186/s40168-023-01618-7

**Published:** 2023-08-07

**Authors:** Xiao-Yu Zhu, Yang Li, Chun-Xu Xue, Ian D. E. A. Lidbury, Jonathan D. Todd, David J. Lea-Smith, Jiwei Tian, Xiao-Hua Zhang, Jiwen Liu

**Affiliations:** 1 Frontiers Science Center for Deep Ocean Multispheres and Earth System, College of Marine Life Sciences, Ocean University of China, 5 Yushan Road, Qingdao, 266003 China; 2 Laboratory for Marine Ecology and Environmental Science, Laoshan Laboratory, Qingdao, 266273 China; 3 Institute of Evolution & Marine Biodiversity, Ocean University of China, Qingdao, 266003 China; 4 School of Biological Sciences, University of East Anglia, Norwich Research Park, Norwich, NR4 7TJ UK; 5 Molecular Microbiology: Biochemistry to Disease, School of Biosciences, The University of Sheffield, Sheffield, S10 2TN UK; 6 Key Laboratory of Physical Oceanography, Ministry of Education, Ocean University of China, Qingdao, 266100 China

**Keywords:** Hadal trench, Metagenomics, Sinking debris, Cell wall polysaccharide utilization, *Bacteroidetes*

## Abstract

**Background:**

Hadal trenches (>6000 m) are the deepest oceanic regions on Earth and depocenters for organic materials. However, how these enigmatic microbial ecosystems are fueled is largely unknown, particularly the proportional importance of complex polysaccharides introduced through deposition from the photic surface waters above. In surface waters, *Bacteroidetes* are keystone taxa for the cycling of various algal-derived polysaccharides and the flux of carbon through the photic zone. However, their role in the hadal microbial loop is almost unknown.

**Results:**

Here, culture-dependent and culture-independent methods were used to study the potential of *Bacteroidetes* to catabolize diverse polysaccharides in Mariana Trench waters. Compared to surface waters, the bathypelagic (1000–4000 m) and hadal (6000–10,500 m) waters harbored distinct *Bacteroidetes* communities, with *Mesoflavibacter* being enriched at ≥ 4000 m and *Bacteroides and Provotella* being enriched at 10,400–10,500 m. Moreover, these deep-sea communities possessed distinct gene pools encoding for carbohydrate active enzymes (CAZymes), suggesting different polysaccharide sources are utilised in these two zones. Compared to surface counterparts, deep-sea *Bacteroidetes* showed significant enrichment of CAZyme genes frequently organized into polysaccharide utilization loci (PULs) targeting algal/plant cell wall polysaccharides (i.e., hemicellulose and pectin), that were previously considered an ecological trait associated with terrestrial *Bacteroidetes* only. Using a hadal *Mesoflavibacter* isolate (MTRN7), functional validation of this unique genetic potential was demonstrated. MTRN7 could utilize pectic arabinans, typically associated with land plants and phototrophic algae, as the carbon source under simulated deep-sea conditions. Interestingly, a PUL we demonstrate is likely horizontally acquired from coastal/land *Bacteroidetes* was activated during growth on arabinan and experimentally shown to encode enzymes that hydrolyze arabinan at depth.

**Conclusions:**

Our study implies that hadal *Bacteroidetes* exploit polysaccharides poorly utilized by surface populations via an expanded CAZyme gene pool. We propose that sinking cell wall debris produced in the photic zone can serve as an important carbon source for hadal heterotrophs and play a role in shaping their communities and metabolism.

Video Abstract

**Supplementary Information:**

The online version contains supplementary material available at 10.1186/s40168-023-01618-7.

## Introduction

The ocean water column is composed of the epipelagic (0–200 m), mesopelagic (200–1000 m), bathypelagic (1000–4000 m), abyssopelagic (4000–6000 m), and hadopelagic/hadal (>6000 m) layers. Due to their extreme depth, the hadal regions are the least-explored aquatic biosphere on Earth and are almost exclusively composed of trenches [1]. The Mariana Trench located in the Western Pacific is the deepest trench on Earth with a maximum depth of ~11,000 m in the Challenger Deep. Apart from permanent darkness, hadal organisms have to cope and adapt to the ultrahigh hydrostatic pressure (up to ~110 Mpa) and stable low temperature (~2 °C) that characterize this ecosystem [1, 2]. Microbial metabolism in the hadal ocean is primarily heterotrophic, sustained by organic matter from sunlit surface waters deposited via sinking particles (e.g., phytoplankton aggregates and zooplankton fecal pellets) and animal carcasses [1, 3, 4]. Other supplies of organic carbon from terrestrial sources and in situ chemosynthetic production are also likely significant [5–8]. Compared to adjacent abyssal plains and slopes, increased microbial cell abundance and respiration rates have been detected in hadal trench sediments [9, 10]. This is likely due to the unique funnel-shaped topography of hadal trenches, which accelerates the collection of organic matter at the trench bottom [3], effectively making them depocenters [7]. Due to the extensive degradation of labile compounds during vertical deposition, microbial heterotrophs in the hadal ocean are thought to contain unique catabolic capacities to utilize the unpalatable “leftovers”. Supporting this, pioneering studies of hadal waters and sediments in the Mariana Trench have reported the enrichment of microbes involved in recycling complex macromolecules, hydrocarbons, and some heavy metals like arsenic and selenium [11–15]. However, the detailed metabolic properties of dominant microbial heterotrophs inhabiting the hadal ocean, such as *Bacteroidetes*, are still poorly understood.

*Bacteroidetes* were found to be more abundant in hadal waters than in overlying abyssopelagic/bathypelagic waters of the Mariana [2], Japan [16], and Kermadec Trenches [17], suggesting these bacteria may play crucial roles in hadal carbon cycling. *Bacteroidetes* are one of the most abundant phyla in the global surface ocean [18], and its members are assumed to be specialized in degrading high molecular weight compounds, especially polysaccharides [19]. In marine ecosystems, polysaccharides are energy storage products and cell wall constituents in phytoplankton and macroalgae, representing a large fraction of their biomass [20]. Correspondingly, abundant *Bacteroidetes*, particularly members of class *Flavobacteriia*, are frequently detected during and after phytoplankton blooms [21–23]. Carbohydrate-active enzymes (CAZymes), such as glycoside hydrolases (GHs) and polysaccharide lyases (PLs), are key enzymes required for polysaccharide degradation. CAZymes encoded in the genomes of *Bacteroidetes* are usually colocalized into gene clusters termed polysaccharide utilization loci (PULs), which also encode for polysaccharide binding and uptake genes. Thus, in addition to numerous CAZymes, a typical PUL contains genes coding for a glycan-binding protein and a TonB-dependent transporter, named Starch Utilization System (Sus) components D and C, respectively, as well as various accessory proteins such as peptidases (targeting glycoproteins), sulfatases (targeting sulfated polysaccharides) [24], and recently reported phosphatases (targeting phosphorylated polysaccharides) [25]. CAZymes display exquisite specificity that distinguishes the carbohydrate moiety and type of glycosidic bond; hence, the CAZyme gene composition within a PUL can provide information on the structure of its targeted polysaccharide [19, 21, 23]. *Bacteroidetes* inhabiting different ecological niches with different substrate availability are often equipped with distinct PUL reservoirs [26–28] with laterally acquired CAZyme genes playing a potentially important role in their niche expansion [29–31].

Here, we hypothesized that hadal *Bacteroidetes*, relative to their surface water counterparts, specialize in the recycling of semi-labile polysaccharides that likely accumulate in deep trenches. To test this hypothesis, multi-omics and cultivation-based methods were used to compare the polysaccharide utilization potentials of *Bacteroidetes* from different layers of the Mariana Trench, as a window to trace the ecological role of these key heterotrophs in the hadal ocean. Our results reveal that the hadal zone harbors an abundant and unique *Bacteroidetes* population with significantly enhanced polysaccharide metabolic potential, especially for degrading cell wall hemicelluloses and pectins likely derived from algae and land plants.

## Results and discussion

### Bathypelagic and hadal *Bacteroidetes* communities differ from those in the surface ocean

**Fig. 1:**
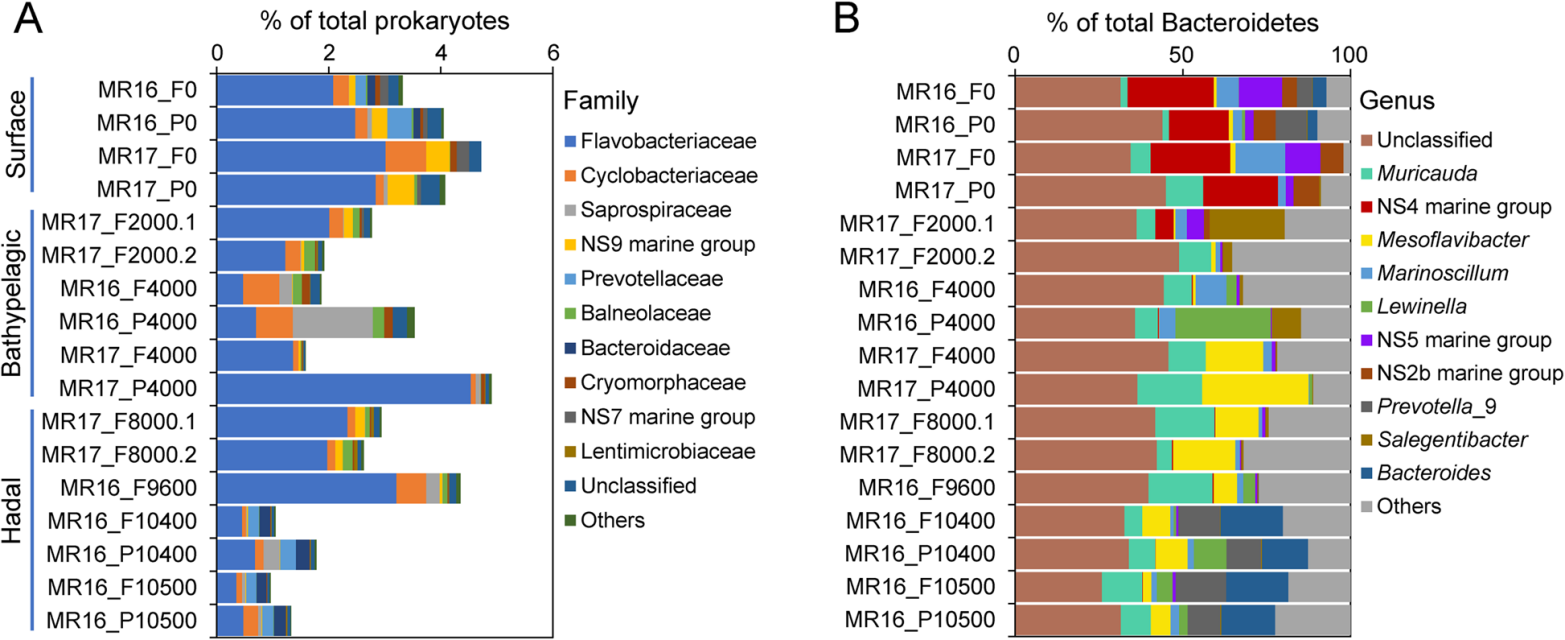
Metagenome-derived relative abundance (16S rRNA gene) of *Bacteroidetes* at family (**A**) and genus (**B**) levels. Sample names were defined by size fraction, sampling depth, and sampling year, e.g., MR16_P0 was the particle-associated sample from 0-m seawater collected in 2016. F, free-living (0.2–3 µm); P, particle-associated (>3 µm). Samples were grouped by depth (surface: 0 m; bathypelagic: 2000 and 4000 m; hadal: 8000, 9600, 10,400, and 10,500 m)

Seventeen seawater metagenomes spanning different water depths from 0 to 10,500 m (Table S1), collected from our previously published studies, were analyzed [6, 11]. To determine the depth profile of *Bacteroidetes* communities in the Mariana Trench, 16S rRNA gene reads were extracted and classified from the metagenomes (Fig. 1). In agreement with previous reports [32], *Bacteroidetes* showed a higher relative abundance in particle-associated (>3 µm) than free-living (0.2–3 µm) samples at most depths (Fig. 1A). The highest relative abundances of *Bacteroidetes* (mainly *Flavobacteriaceae* family) were recorded at 0, 4000, and 9600 m, comprising up to 5% of the total prokaryotic community. The relative abundance of *Bacteroidetes* decreased from surface to 2000 m waters, then increased at 4000–9600 m, and finally decreased in the near-bottom-samples (10,400 and 10,500 m). A similar distribution pattern was described in a previous study of the Mariana Trench [2], but with a greater abundance of *Bacteroidetes* as a percentage of the hadal prokaryotic community in surface and ≥ 6000 m samples (Fig. S1)*.* Despite this difference in relative abundance, both studies demonstrate *Bacteroidetes* can constitute a significant fraction of the surface and hadal water microbial communities.

Distinct communities of *Bacteroidetes* genera were observed at different depths (Fig. 1B, Fig. S2). Strikingly, *Mesoflavibacter* was enriched at ≥ 4000 m, whilst the relative abundance of NS4/NS5/NS2b marine groups and *Marinoscillum* was associated with surface waters. Most *Bacteroidetes* isolates from a previous cultivation-dependent study of Mariana Trench hadal waters were *Mesoflavibacter* and *Muricauda*, consistent with their high abundance in these deep water environments [33]. Intriguingly, we observed a significant relative enrichment of *Prevotella* and *Bacteroides*, known to be the main anaerobes of human gut and fiber-rich rumen involved in degrading plant polysaccharides for their hosts [34–36], in the aerobic near-bottom waters (Fig. 1B). We speculate that these two genera might be attached to sinking organic aggregates, where concentrated respiration results in local O_2_ exhaustion [37].

### Distinct *Bacteroidetes* CAZyme gene pools are present in bathypelagic and hadal waters

**Fig. 2:**
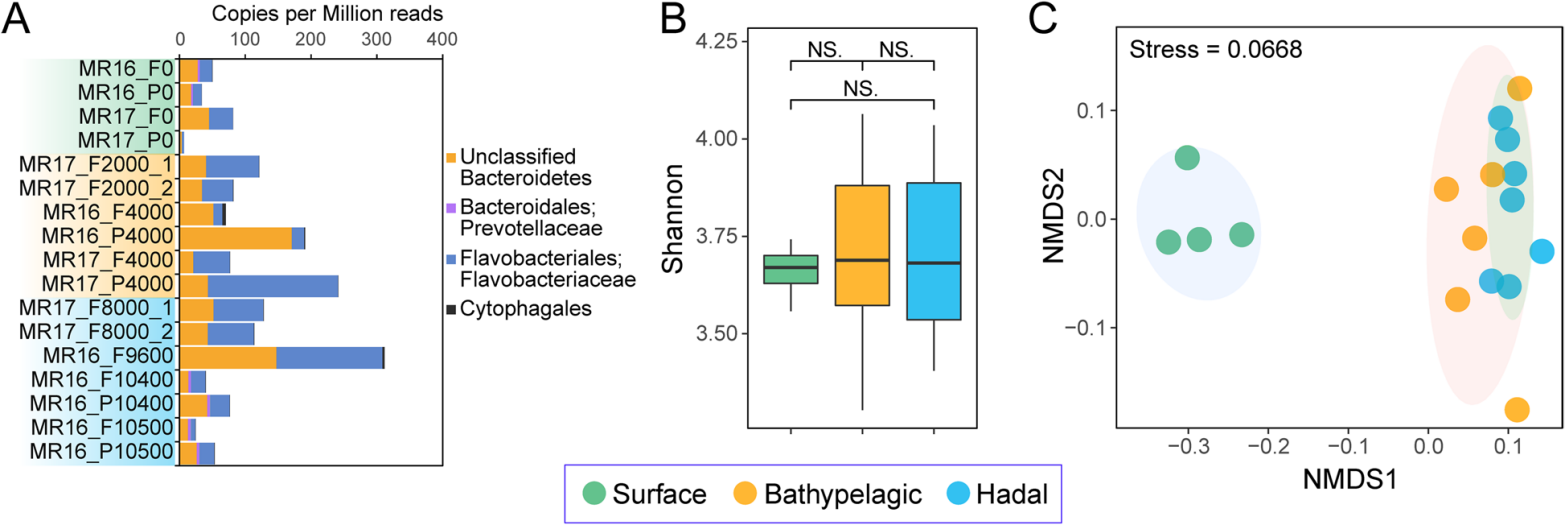
Comparison of *Bacteroidetes* CAZyme genes at different water depths of the Mariana Trench. **A** Relative abundance (copies per million reads, CPM) of *Bacteroidetes* CAZyme genes at each depth. **B** Shannon diversity of *Bacteroidetes* CAZyme genes in the surface, bathypelagic and hadal zones. Statistics were based on the Wilcoxon test. NS., no significance. **C** Non-metric Multi-Dimensional Scaling (NMDS) analysis based on Bray-Curtis dissimilarities of the composition of *Bacteroidetes* CAZyme genes in each sample. The shaded ellipses represent the 80% confidence interval

Given the specialized ability of *Bacteroidetes* to degrade polysaccharides [25], we investigated their CAZyme genes across water depths (Fig. 2, Table S2). Most of these genes were affiliated to *Flavobacteriaceae.* Whilst the importance of *Bacteroidetes-*mediated polysaccharide cycling has been well documented in surface waters [21, 23, 38, 39], we observed that the relative abundance of *Bacteroidetes* CAZyme genes generally increased with depth, except for a large decrease in near-bottom waters (Fig. 2A). This suggests *Bacteroidetes* may also significantly impact deep-sea carbon cycling. Whilst the alpha diversity (Shannon index) of *Bacteroidetes* CAZyme genes did not significantly differ between water layers (Fig. 2B), the overall composition of CAZyme gene pools differed significantly (ANOVA, *p* < 0.05) between bathypelagic/hadal (hereafter BH) and surface waters (Fig. 2C). This included the GH/PL (two representative degradative CAZyme families) gene pools (Fig. S3; ANOVA, *p* < 0.05) and was concordant with the distinct community composition between these two zones (Fig. 1, Fig. S2; ANOVA, *p* < 0.05), reflecting taxa dependent CAZyme variations. As CAZyme gene reservoirs likely reflect biogeographic patterns of carbohydrate supply [40], our results suggest distinct polysaccharide pools exist in the surface and BH oceans of the Mariana Trench, consistent with differences observed in other regions in the global ocean [41].

### Increased polysaccharide metabolic versality of BH *Bacteroidetes*

**Fig. 3:**
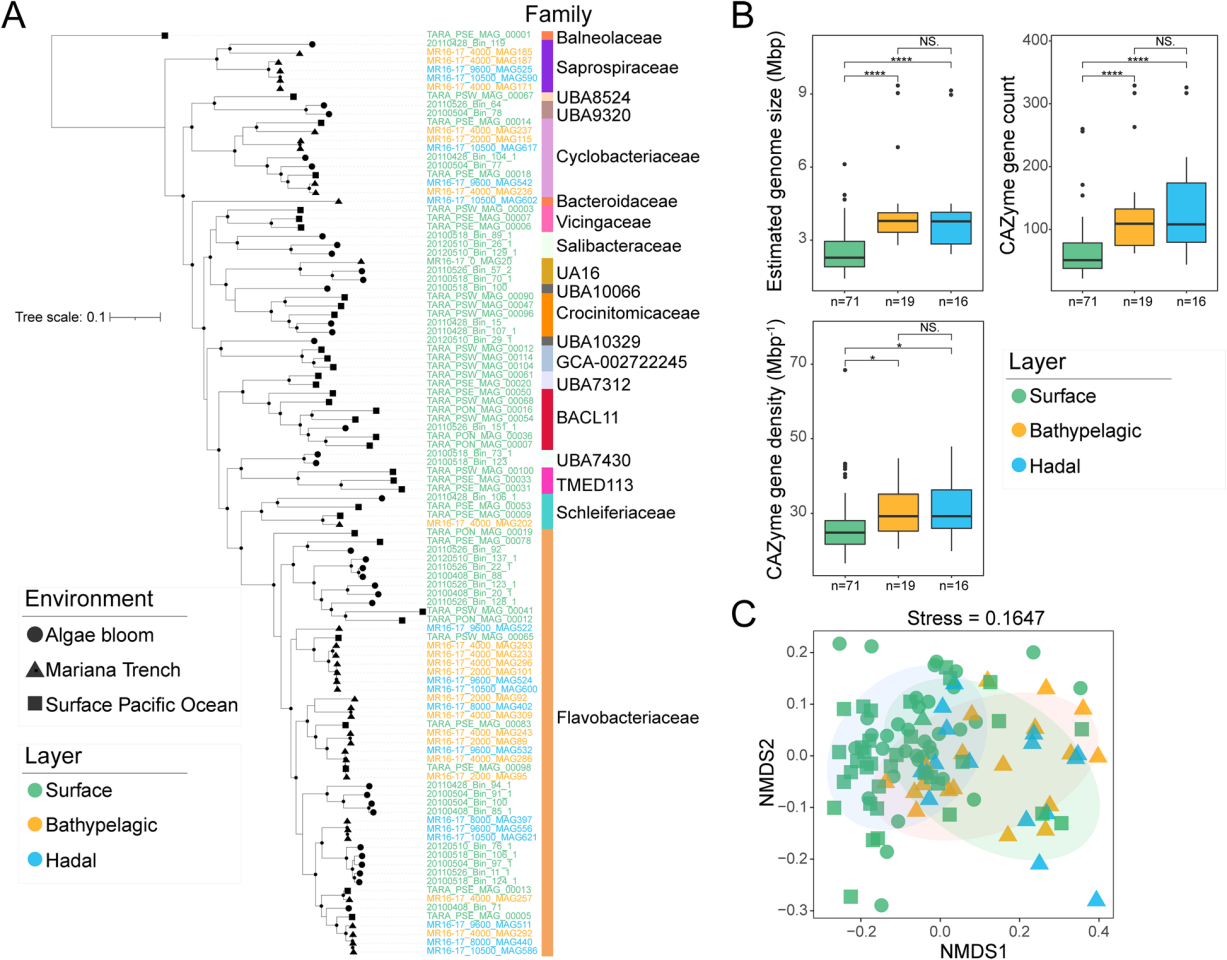
*Bacteroidetes* MAGs used in this study, their phylogeny and genome comparisons. **A** Phylogenomic tree of *Bacteroidetes* MAGs based on 120 conserved single-copy genes identified by GTDB-Tk v1.7.0 [44]. Genomes were grouped according to their isolated environments (shapes) and isolated water layers (colors). Bootstraps > 70% are indicated by black nodes. Tree scale indicates evolutionary distance as the rate of substitution per site. **B** Comparison of estimated genome size, CAZyme gene count, and CAZyme gene density among surface (*n* = 71), bathypelagic (*n* = 19), and hadal (*n* = 16) MAGs. Statistics were based on the Wilcoxon test. *p < 0.05; ****p < 0.0001; NS., no significance. **C** Bray-Curtis dissimilarities of *Bacteroidetes* MAGs illustrated by NMDS analysis based on the composition of CAZyme genes in each MAG. The shaded ellipses represent the 80% confidence interval

We further explored the difference in potential polysaccharide substrates between surface and BH *Bacteroidetes* MAGs by analyzing their PUL and CAZyme gene contents. A total of 36 *Bacteroidetes* MAGs were recovered from all depths [42], including one from the surface, 19 from the bathypelagic and 16 from the hadal zone (Table S3). To enable comparisons between surface and BH MAGs, we analyzed additional MAGs (*n *= 35) from surface seawaters of the Pacific Ocean (*Tara Ocean* project) [43] and those (*n *= 35) from diatom blooms in the North Sea (Table S3), where algae polysaccharides stimulated abundant *Bacteroidetes* [21]. Most of these MAGs (86%, 92/106) had >75% completeness and <5% contamination. Phylogenomic analysis indicated that these MAGs covered a broad range of *Bacteroidetes* families (Fig. 3A). Estimation of the relative abundance of these MAGs indicated that they were almost always specific to the environment they were recovered from (Fig. S4, Table S4), implying niche partitioning of marine *Bacteroidetes* within closely related groups. The average number of CAZyme genes in BH MAGs (129.7 genes and 135.8 genes) were almost twice those of MAGs retrieved from surface waters (65.9 genes). This was not only a result of a larger BH MAG genome size (Wilcoxon, *p* < 0.05) but also of a higher CAZyme gene density (Wilcoxon, *p* < 0.05) (Fig. 3B, Table S3). This highlighted the greater capacity for polysaccharide degradation in BH *Bacteroidetes*. Unlike in the metagenomic samples (Fig. S2), some CAZyme genes and GH/PL gene pools in BH MAGs were not dissimilar to their surface counterparts (Fig. 3C, Fig. S5), possibly due to a lack of phylogenetic separation (Fig. 3A).

### BH *Bacteroidetes* MAGs are enriched in CAZymes targeting hemicelluloses and pectins

**Fig. 4:**
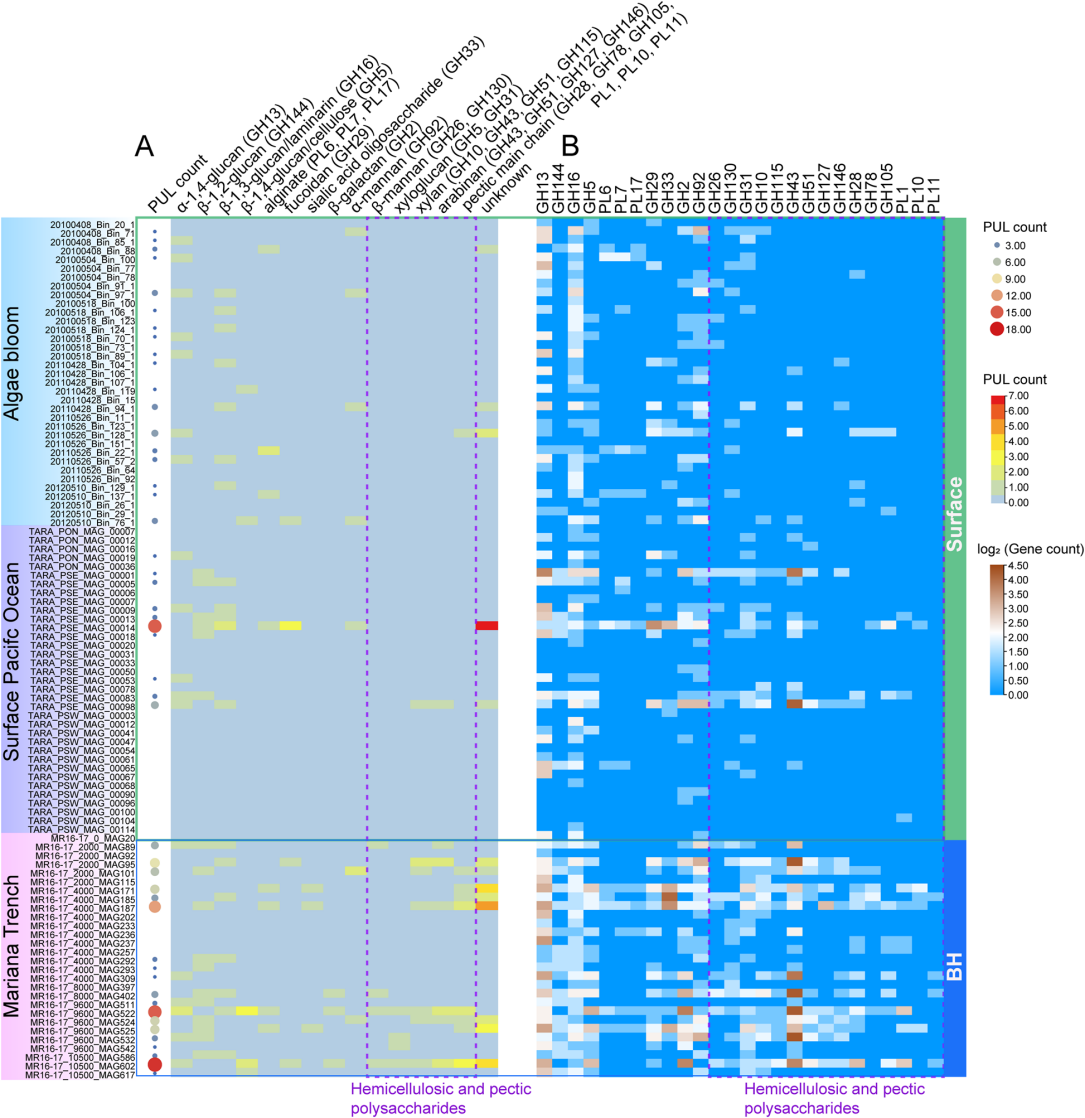
Polysaccharide utilization potential of *Bacteroidetes* MAGs revealed by predicted PULs and related key CAZyme genes. **A** The distribution of predicted PULs in all MAGs. PULs were classified based on their potential substrates. GH or PL families in the bracket represent common genes involved in degrading those glycans. **B** The distribution of key PUL genes in all MAGs. MAGs were grouped by their isolated environments and isolated water layers. BH, bathypelagic and hadal

Habitat is considered as one of the main driving forces of carbohydrate catabolism in marine *Bacteroidetes* [45]*.* Therefore, the enhanced polysaccharide utilization capacity of BH *Bacteroidetes* could have evolved from the requirement to acquire energy from complex organic material in BH zones [4]. To determine polysaccharide utilization differences between populations, PULs were predicted for MAGs to indicate their potential polysaccharide substrates [21, 23]. We employed a stringent PUL prediction criterion, specifically requiring the presence of at least one *susCD* gene pair and at least two degradative CAZyme genes from GH or PL families. Replicated MAGs from each of the three water zones of the Mariana Trench respectively were removed to avoid potential exaggeration. Algal bloom and surface Pacific Ocean MAGs were already non-redundant. Approximately 70% (19 of 27) of the BH MAGs were predicted to contain PULs, far more than from surface MAGs (39%, 28 of 71) (Fig. 4A, Table S5). These PULs were classified into 14 types according to their predicted substrates (Fig. 4A, Table S5). The BH MAGs featured more abundant and diverse PULs compared to their surface counterparts.

Due to the absence of PULs in some MAGs, likely attributed to their highly fragmented genomes hindering PUL prediction (Fig. 4A, Table S5), and the scattered CAZyme genes across the genomes, we also assessed the distribution of the relatively conserved CAZyme genes, considered to be key in the identified intact PULs, in each MAG (Fig. 4B, Table S6). It turned out they were also more widely distributed in BH MAGs (Fig. 4B). PULs/genes predicted to target α-1,4-glucan (GH13 α-amylase/α-glucanase), β-1,3-glucan/laminarin (GH16 β-1,3-glucanase), β-1,4-glucan/cellulose (GH5, a family of mostly β-glucanases), α-mannan (GH92 α-mannosidase), fucose-containing glycan (GH29 α-L-fucosidase), sialic acid oligosaccharides (GH33 sialidase), and β-galactan (GH2, a family of diverse functions) did not show enrichment in surface or BH MAGs (Fig. 4A, B). Conversely, PULs/genes targeting hemicellulosic (xyloglucan, xylan, and β-mannan) and pectic (arabinan and pectic main chain including rhamnogalacturonan-I and homogalacturonan) glycans were largely confined to the BH MAGs.

### CAZyme genes for hemicelluloses and pectins are more abundant in deeper Mariana Trench waters

**Fig. 5:**
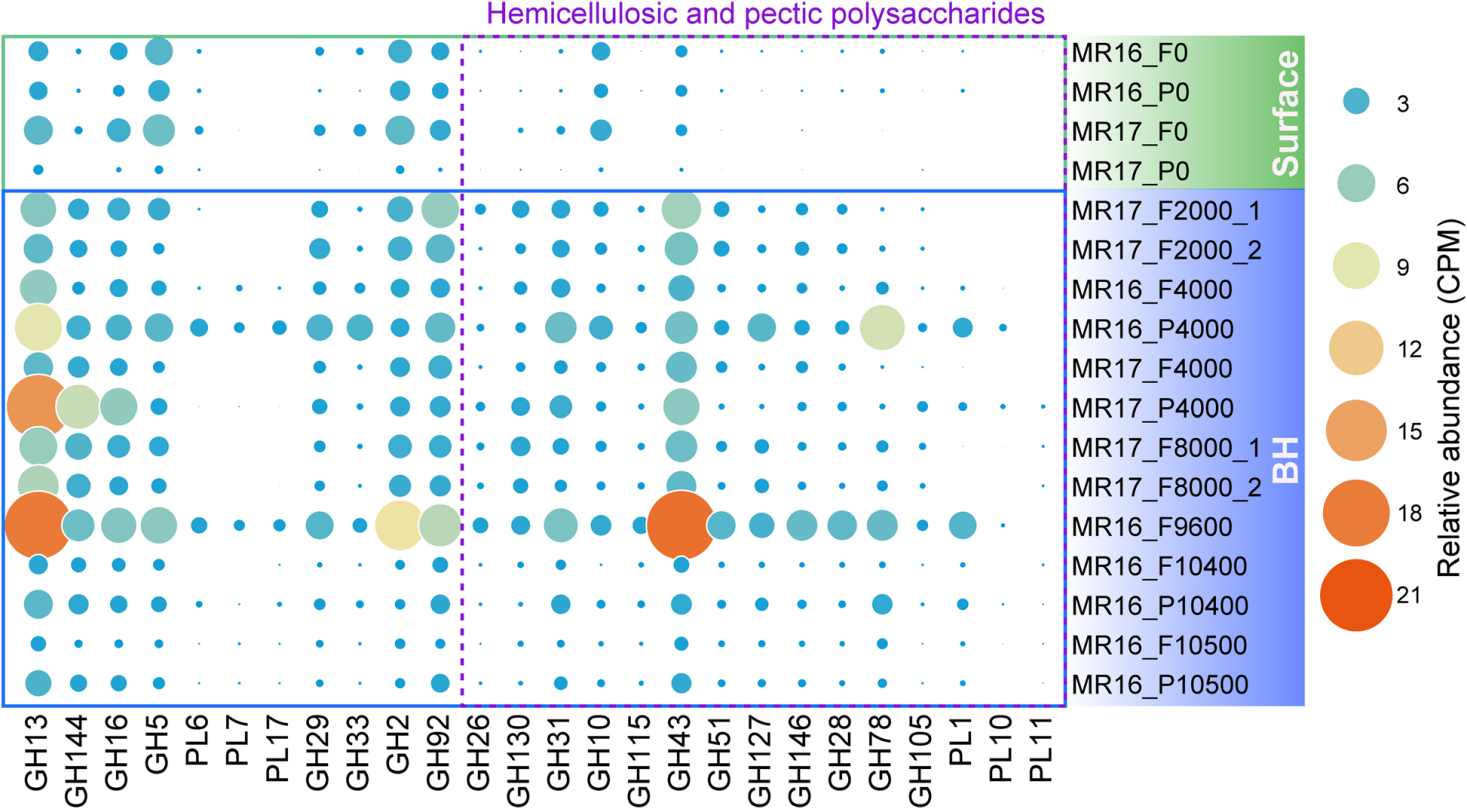
Relative abundance of key *Bacteroidetes* PUL genes across the Mariana Trench water column. Potential substrates for these genes can be seen in Fig. 4. BH, bathypelagic and hadal

Differential analyses based solely on the MAGs we examined may not reliably reflect samples from Mariana Trench surface seawater since all but one surface MAGs were derived from other oceanic regions. Therefore, we further investigated the relative abundance of key PUL genes across the whole Mariana Trench water column using the entire metagenomic dataset (Fig. 5, Table S2). Consistent with the MAG analysis (Fig. 4), genes for hemicellulose and pectin degradation were barely detected (except for xylan degradation, GH10) in surface water metagenomes but were strongly enriched in BH waters (Fig. 5). However, genes coding for GH115 (xylan α-1,2-glucuronidase) were only seen in the BH zones, implying that xylan may only be partially degraded in surface waters and fully degraded below. Pectins and hemicelluloses are major components of plant and macroalgal cell walls [20, 46]. Recently, these polysaccharides were detected in surface waters during a diatom bloom [47] and associated *Bacteroidetes* spp. were shown to possess PULs for their degradation [21, 23, 39]. Since algal cell walls evolved to be highly resistant to a wide range of biotic stresses like enzymatic hydrolysis, their pectic and hemicellulosic glycan components may persist longer than internal storage glycans like laminarin [47] and, thus, be more likely to sink to deeper waters. The BH *Bacteroidetes* of the Mariana Trench may act as “scavengers” recycling the cell wall polysaccharides which are poorly degraded in the upper ocean. This hypothesis is supported by the observation that *Maribellus comscasis*, a deep-sea *Bacteroidetes* [48], has high hemicellulose degradation activity and that organic matter in the Mariana Trench is thought to be primarily from marine algae with minor terrestrial inputs [7]. This aligns with our genomic observations that hadal *Bacteroidetes* possess wider polysaccharide degradation potential, leading to their significantly enhanced ability to decompose hemicelluloses and pectins. Although the polysaccharide compositions of the analyzed seawater samples are unavailable, the detected CAZymes may indicate the presence of specific polysaccharides [19], which warrants further investigation.

Conversely, genes predicted to encode α-1,4-glucan (GH13), β-1,3-glucan/laminarin (GH16), β-1,4-glucan/cellulose (GH5), α-mannan (GH92), and β-galactan (GH2) degradation enzymes were prevalent in both surface and BH waters (Fig. 5, Table S2), indicating these compounds were likely essential nutritional sources for *Bacteroidetes* at all depths. Genes targeting alginate (PL6/PL7/PL17, alginate lyase) were barely detected in any samples, likely due to alginates being derived from coastal macroalgae and thus being rarely found in pelagic waters. Although PUL gene products likely targeting β-1,2-glucan (GH144 endo-β-1,2-glucanase) were predicted in both surface and BH MAGs (Fig. 4A), they were significantly enriched in BH waters (Wilcoxon test, *p* < 0.05; Fig. 5, Table S2), hinting that β-1,2-glucan is more abundant in BH waters.

### A distinct PUL in hadal localized *Mesoflavibacter* strain MTRN7 enables utilization of the pectic polysaccharide arabinan

**Fig. 6:**
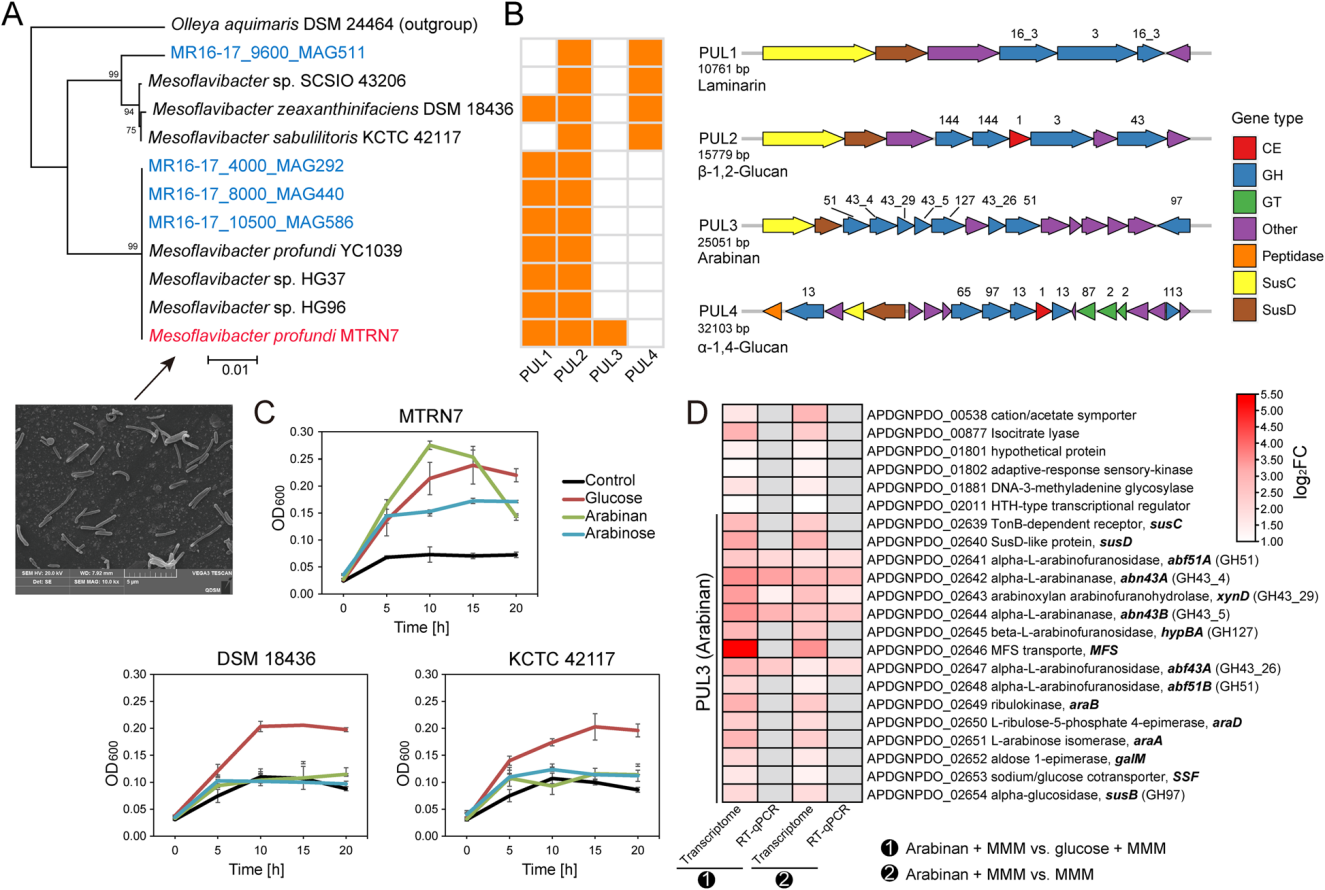
Polysaccharide utilization potential of *M.* *profundi* MTRN7. **A** Phylogenetic inference of MTRN7 based on 120 conserved single-copy genes identified by GTDB-Tk v1.7.0 [44]. Strain MTRN7 is marked in red while recovered MAGs are marked in blue. TEM observation of MTRN7 was shown. **B** Predicted PULs in *Mesoflavibacter* MAGs. **C** Growth assays of MTRN7 and two reference strains (*M. zeaxanthinifaciens* DSM 18436 and *M. sabulilitoris* KCTC 42117) in marine mineral medium (MMM) supplemented with glucose, arabinose, and arabinan at atmospheric pressure. MMM without supplemented sugars or polysaccharides was used as a control. **D** All genes significantly upregulated (|log_2_FC| > 1 and FDR-adjusted *p* < 0.05) when MTRN7 was grown with basal medium plus arabinan compared to basal medium and basal medium plus glucose. Several genes located in the arabinan PUL were further examined by RT-qPCR. Gray bricks indicate genes that were not quantified

To complement our genetic predictions on distinct CAZyme occurrence in BH *Bacteroidetes*, we combined physiological experiments and heterogenous production synthesis experiments to investigate the polysaccharide utilization ability of a *Mesoflavibacter* strain MTRN7 (Fig. 6). MTRN7 was isolated from the Mariana Trench at 8727 m (Table S7) and is closely related (99.7%, 16S rRNA gene identity) to a *Mesoflavibacter profundi* strain (Fig. 6A, Fig. S6) also isolated from the deep sea of the Mariana Trench (Table S7) [49]. As expected, MTRN7 could grow at physiologically relevant pressure (60 MPa, equal to 6000 m) at room temperature on a nutrient-rich 2216E medium (Fig. S7), indicating its strong adaptation to high pressure. Pressure tolerance equivalent to that of the isolation depth (~87 Mpa) was not obtained, possibly due to the limitations of the culture conditions, e.g., lack of potential osmolytes conferring resistance to high hydrostatic pressure.

We identified four PUL types from MTRN7, the four *Mesoflavibacter* MAGs recovered from 4000–10,500 m, and publicly available *Mesoflavibacter* genomes from other environments (Fig. 6B, Table S7). While PULs predicted to target β-1,3-glucan/laminarin, β-1,2-glucan and α-1,4-glucan were relatively common in most *Mesoflavibacter* strains, the PUL targeting the pectic polysaccharide arabinan was unique in MTRN7. The predicted arabinan PUL showed a consistent distribution with *Mesoflavibacter* genera across the water column (Fig. 1B, Fig. S8), indicating that MTRN7 is an important member of *Mesoflavibacter* in the Mariana Trench. Supporting the genomic analysis, only MTRN7 and not two surface-derived *Mesoflavibacter* strains (DSM 18436 and KCTC 42117) lacking the predicted arabinan PUL could use arabinan and arabinose as carbon sources (Fig. 6C). Compared to no sugar and glucose, 21 genes were upregulated when supplied with arabinan (Fig. 6D). All genes in this PUL were strongly activated during growth on arabinan, strongly suggesting the involvement of this PUL in utilizing this polysaccharide (Fig. 6D). This PUL features an arabinoxylan arabinofuranohydrolase (XynD), two alpha-L-arabinanases (Abn43A and Abn43B), three *α*-L-arabinofuranosidases (Abf51A, Abf43A, and Abf51B) and one *β*-L-arabinofuranosidase (HypBA) (Fig. 6D, Fig. S9). These enzymes are crucial in breaking down arabinan into arabinose [51, 52]. In contrast to previously reported PULs for arabinan degradation [51, 52], the newly found arabinan PUL in MTRN7 also encoded enzymes predicted to convert arabinose to xylulose-5-P, which can enter central metabolism (Fig. S9). Therefore, this arabinan PUL likely confers on MTRN7 a complete arabinan metabolism pathway.

### Arabinan utilization in MTRN7 appears horizontally acquired from a coastal *Flavobacteriaceae* strain

**Fig. 7:**
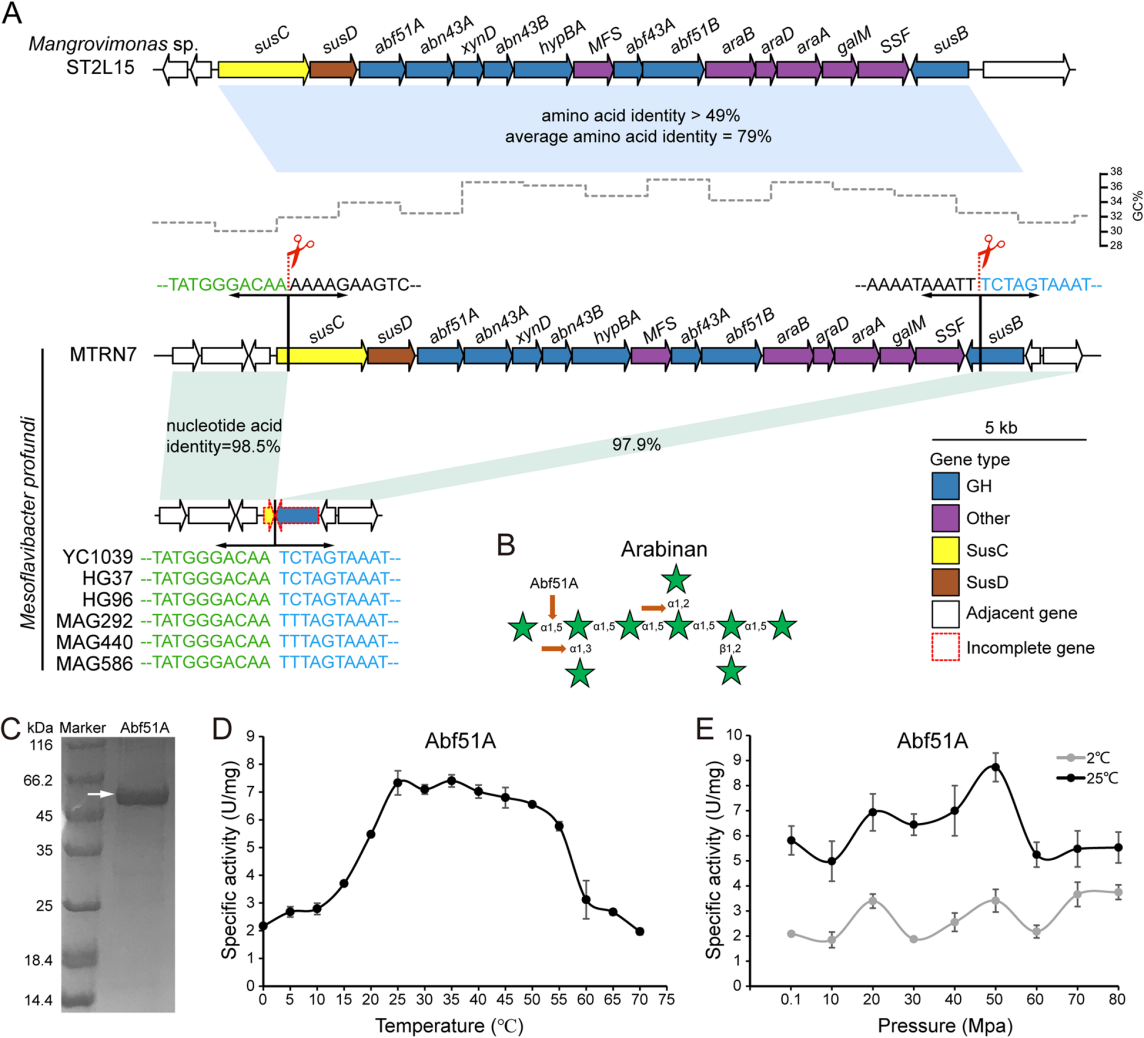
The acquired arabinan PUL of *M. profundi* MTRN7. **A** Schematic depiction of two similar arabinan PULs found in the mangrove (seashore) strain *Mangrovimonas* sp. ST2L15 and the hadal strain *M. profundi* MTRN7. Genes are shown to scale and colored according to putative function. The complete name of each gene is listed in Table S3. Light blue bars indicate amino acid sequence identities over 40% while light green bars indicate nucleotide acid sequence identities over 97%. Comparison of the MTRN7 PUL with other genomes of *M. profundi* (e.g., YC1039) detailing conserved regions in the start (*susC*) and end (*susB*) genes. The red scissors indicate the sites between which the remaining PUL was lost. The GC content of MTRN7 is shown above the genes and was calculated using a non-overlapping sliding window of 2000 bp. **B** Predicted cleavage sites of an α-L-arabinofuranosidase (Abf51A) from the MTRN7 arabinan PUL. **C** Purification of Abf51A. The specific activity of Abf51A on *p*NP-α-L-arabinofuranose was tested under a range of temperatures from 0 to 70 °C (**D**) and pressures from 0.1 to 80 Mpa at 2 °C and 25 °C (**E**)

To address why the arabinan PUL was only observed in MTRN7, we performed a comparative genomic analysis of six other *M. profundi* strains against MTRN7. Interestingly, a deletion event between the start (*susC*) and end (*susB*) genes was observed in all these strains, with strong conservation of the remaining upstream and downstream regions (>97.8% nucleotide acid identity) (Fig. 7A). This suggests that the arabinan PUL was likely present in all those *M. profundi* genomes but only MTRN7 retained this gene cluster. More interestingly, the predicted arabinan PUL had a higher GC content (34.3%) compared to that of the whole MTRN7 genome (31.2%) (Fig. 7A). This implies the arabinan PUL previously originated from a horizontal transfer event. BLASTp analysis against the NCBI nr database revealed that proteins from this predicted arabinan PUL had the highest homology (amino acid identities 49–93%, 79% on average) with proteins from a PUL with the same gene content and gene order identified in another *Flavobacteriaceae* strain, *Mangrovimonas* sp. ST2L15 (Fig 7A, Table S8), which was isolated from a mangrove forest located at the land-sea boundary in Malaysia [53]. The arabinan PUL similarity between MTRN7 and ST2L15 (79% on average) was also much greater than the average amino acid similarity of all other gene pairs from the two genomes (61.8% on average). Unlike in MTRN7, the GC content (37.3%) of the arabinan PUL in ST2L15 was similar to the whole genome (36.2%). A phage integrase, specifically a tyrosine recombinase (gene id: APDGNPDO_02609), is located about 30 genes upstream of the arabinan PUL and could be responsible for the transfer. Similar phage integrases have also been reported around two other laterally acquired PULs and have been suggested as important elements for the integration of external PUL into genomic DNA [29, 30]. Finally, through phylogenetic analyses, we validated that the MTRN7 arabinan PUL was likely acquired from coastal/land *Bacteroidetes* (see supplementary data for detailed results, Fig. S10).

Considering the potential coastal/terrestrial origin of the arabinan PUL, we next determined whether the MTRN7 CAZymes have evolved to function in hadal extreme conditions (e.g., low temperature and high pressure). To address this, a secretory *α*-L-arabinofuranosidase (Abf51A) predicted to cleave multiple linkages (*α*-1,2, *α*-1,3, and *α*-1,5) of purified arabinan within the PUL was heterogeneously expressed (Fig. S9, Fig. 7B, C) and examined for activity from 0 to 70 °C (Fig. 7C). Abf51A had an optimum temperature range of 25–35 °C and maintained about 30% of the highest activity under low temperatures (0–10 °C). Furthermore, Abf51A was shown to be resistant to high pressures, functioning up to 80 Mpa (Fig. 7D). These findings are supported by our growth experiments demonstrating that MTRN7 can utilize arabinan as a carbon source under high pressure and low temperature (Fig. S11).

### A proposed model of polysaccharide cycling in hadal trenches

**Fig. 8:**
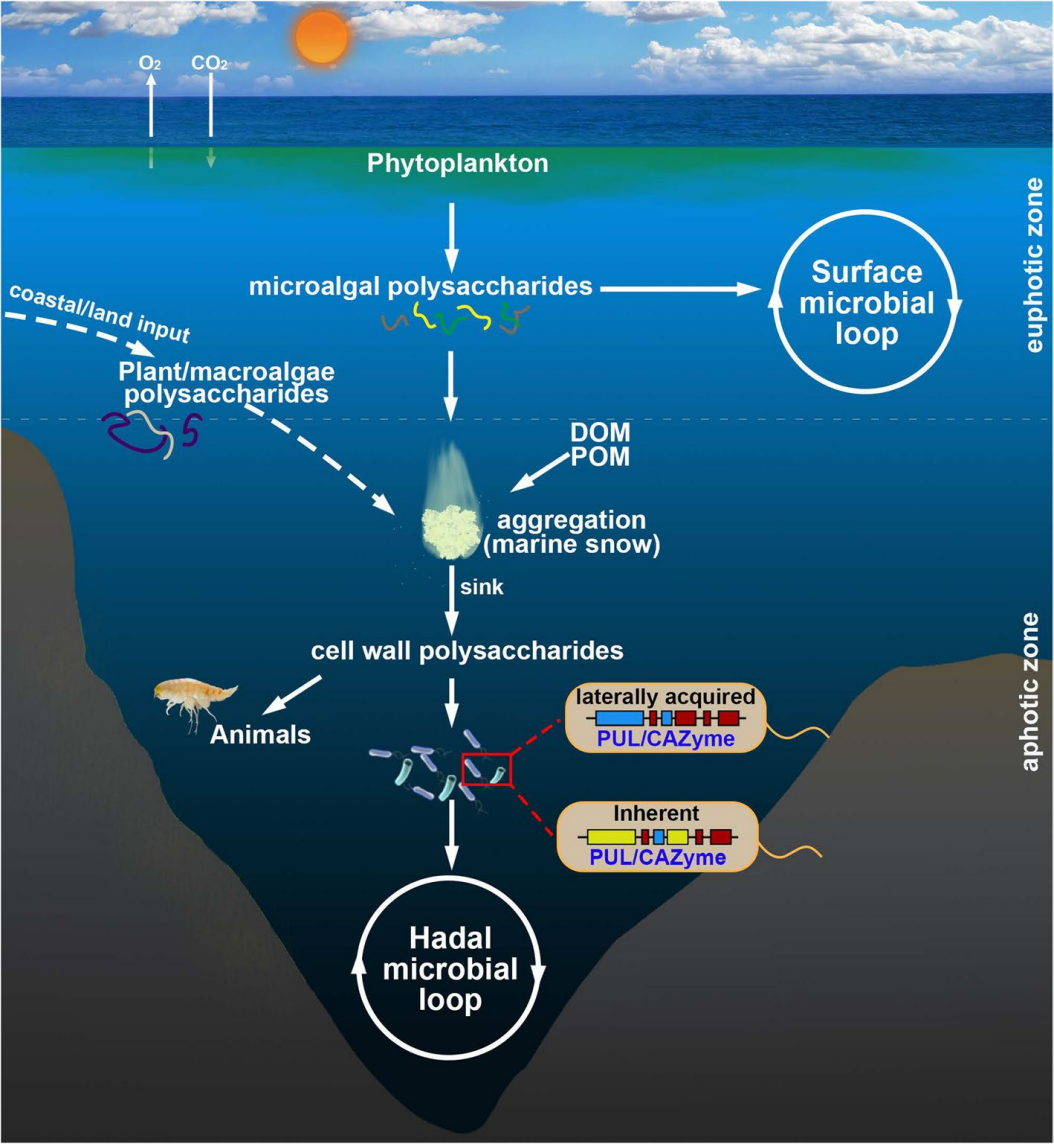
Schematic of potential polysaccharide cycling across the full-water depth of the Mariana Trench. Both phytoplankton and potential coastal/land-derived polysaccharides likely support hadal microorganisms and animals. After the extensive degradation of most liable storage polysaccharides in upper waters during long-distance descent, abundant cell wall polysaccharides are left in the hadal zone. These cell wall polysaccharides are initially consumed by specialized polysaccharide-degrading organisms like *Bacteroidetes* with possibly laterally acquired PULs. DOM, dissolved organic matter; POM, particulate organic matter

Since most of the deep ocean remains unexplored, we know comparatively little about the polysaccharide-degrading ability of microorganisms inhabiting this environment [20, 48]. In this study, we utilized metagenomics to investigate the potential polysaccharide sources across the full depth of the Mariana Trench to further our understanding of hadal carbon cycling. Based on our findings, we proposed a model for polysaccharide cycling in the Mariana Trench (Fig. 8). Polysaccharide sources in the Mariana Trench waters are likely predominantly derived from phytoplankton biomass (especially dinoflagellates) [54] with potential coastal and land inputs [7]. After the utilization of labile forms like glucans during the sinking process, structurally complex cell wall polysaccharides including hemicelluloses and pectins accumulate in BH waters, providing an important carbon source for local heterotrophic microorganisms [2, 12, 13, 15] and animals [1, 55]. Genetic elements both inherently present and laterally transferred confer hadal microorganisms with specialized polysaccharide decomposition capabilities. Overall, our data suggests that specialized *Bacteroidetes* spp. play an integral role in hadal carbon cycling akin to surface carbon cycling [56], but with a preference in degrading cell wall polysaccharides.

## Materials and methods

### Data collection

Metagenomic data from different depths (0–10,500 m) of the Mariana Trench were obtained from our recent work [6, 11, 57] (Table S1). Water at each depth was fractionized into particle-associated (>3 µm) and free-living (0.2-3 µm) samples. Due to insufficient DNA quality, particle-associated samples from 2000, 8000, and 9600 m were not sequenced successfully. A total of 106 *Bacteroidetes* MAGs were collected in this study, including 36 MAGs from the Mariana Trench, 35 MAGs from mid-bloom and late-bloom stages of spring diatom blooms in the southern North Sea [21] and 35 MAGs from the surface Pacific Ocean [43] (Table S3). To estimate the relative abundance of each MAG in its in situ environment, the related metagenomic data were also downloaded (Table S1).

### Metagenomic data processing

Raw data were first quality trimmed with Trimmomatic v0.36 [58] with options ‘SLIDINGWINDOW:4:15 LEADING:3 TRAILING:3 MINLEN:75’ and then assembled with MEGAHIT v1.0.2 [59] using the default parameters. Assembled contigs were used to predict genes using Prodigal [60] with the “-meta” option. Gene sequences derived from all the metagenomes were combined and subjected to CD-HIT (>95% sequence identity and >90% coverage) to generate a nonredundant gene catalog. Then, estimated relative abundance (CPM, copies per Million mapped reads) of nonredundant genes based on paired-end reads were determined by CoverM (v0.6.1, https://github.com/wwood/CoverM). Briefly, the copy number of each gene was calculated by the “jgi_summarize_bam_contig_depths” module integrated in CoverM and was further normalized by total mapped reads of each metagenome. CAZymes were annotated using run_dbCAN v3.0.5 [61] with the hmmer and diamond modules. Only CAZymes identified by both methods were considered. Taxonomic assignment of each gene was performed with MEGAN v6.21.7 [62]. The community profile was generated by Phyloflash [63] based on extracted 16S rRNA gene reads. Alpha-diversity (Shannon) and beta-diversity (NMDS) analyses of *Bacteroidetes* community and CAZymes composition in different samples were conducted with the “vegan” package in R [64].

### MAG analyses

Genome completeness and contamination were estimated by CheckM v1.0.12 [65]. Taxonomic assignment of each MAG was determined by the “classify” module of GTDB-Tk v1.7.0 [44] based on 120 bacterial marker genes. A maximum-likelihood phylogenetic tree based on concatenated 120 marker genes from the output of GTDB-Tk v1.7.0 [44] was determined by FastTree v2.1.10 (options—gamma) [66] and visualized in the interactive Tree of Life (iTOL) [50]. The relative abundance (percent of mapped reads) of MAGs was calculated using CoverM (v0.6.1, https://github.com/wwood/CoverM), which mapped metagenomic sequences to MAGs with default parameters.

Gene prediction and annotation were performed with Prokka v1.12 [67]. Annotations of CAZymes were performed as described above. Peptidases and sulfatases were respectively annotated by BLASTp searches against the MEROPS v12.1 [68] and the SulfAtlas v1.0 [69] databases with <1e−5 *E* value, >30% identity and >70% subject coverage. The *susC*-like and *susD*-like genes were identified according to their specific Pfam domains (PF00593, PF13715, and PF07715 for *susC*; PF07980, PF12741, PF12771, and PF14322 for *SusD*). PULs were predicted manually based on the presence of tandem *susCD*-like pairs and at least two degradative CAZyme genes from the GH or PL families using a seven-gene sliding window, which is more strict than a previous study [70]. The putative substrate of each PUL was predicted based on the functional annotation of PUL genes and further crossed-checked with experimentally validated PULs in dbCAN-PUL [71]. Relative abundance (RPKM, reads per kilobase per million mapped reads) of the arabinan PUL in each sample was estimated by CoverM (v0.6.1, https://github.com/wwood/CoverM).

### Isolation, substrate utilization, and high-pressure adaptation of *M. profundi* MTRN7

*M. profundi* MTRN7 was isolated at 8727 m depth in the Mariana Trench by culturing in oligotrophic R2A medium. Cell morphology was observed by transmission electron microscopy (JEM-1200EX, JEOL) after cells had been negatively stained with 1 % (w/v) phosphotungstic acid following culturing on marine 2216E agar at 28 °C for 24 h.

Substrate utilization assays of strain MTRN7 and two reference strains (DSM 18436 and KCTC 42117) were performed at atmospheric pressure. Briefly, 1 ml MTRN7 culture (OD_600_=0.5) was inoculated in 50 ml MMM (Table S9) supplied with or without (control) sugars or carbohydrates: 5 g/L glucose, 5 g/L arabinan, and 5 g/L arabinose, at 28 °C for 20 h. Components of MMM are listed in Table S11. An ultraviolet spectrophotometer was used to monitor the growth of MTRN7 every five hours. All assays were performed in triplicate.

To explore its endurance to high pressure, triplicate cultures of the isolates (50 μl, OD_600_=0.8) were added to 450 μl of fresh 2216E medium, transferred into 1-mL sterile syringes, and then sealed with a lid. Syringes were placed in stainless steel reactors (Nantong Feiyu Oil Science and Technology Exploitation, China) and pressurized to 0.1, 10, 20, 40, 60, and 80 MPa at room temperature for 7 days. Bacterial colonies were counted by spreading serially diluted cultures on 2216E plates at 28 °C for 24 h.

To explore the utilization of arabinan under high-pressure and low-temperature conditions, triplicate cultures of the isolates (50 μl, OD_600_=0.8) were added to 450 μl of MMM supplied with a high concentration of arabinan (20 g/L) and incubated under different pressure conditions (i.e., 0.1, 10, 20, 30, 40, and 50 Mpa) at 2 °C. Considering MTRN7 appears to be unable to grow with nutrient-rich 2216E medium above 60 Mpa, we utilised a maximum pressure of 50 Mpa when cultured with arabinan. High-pressure incubation and bacterial colony counting were performed under the same conditions mentioned above.

### Comparative genomics of MTRN7

The genome of MTRN7 was sequenced using the PacBio RS II and Illumina HiSeq 4000 platforms at Majorbio Biopharm Technology Co., Ltd. (Shanghai, China) and assembled by combining the Illumina short reads and the PacBio long reads using Unicycler v0.4.8 [72] with the default parameters. For comparative genome analysis, phylogenetically closed genomes of *Mesoflavibacter* were downloaded from the NCBI database (Table S7). Quality estimation, genome annotation, PUL prediction, and phylogenomic analysis of *Mesoflavibacter* genomes were performed as described above. To reconstruct the phylogenetic tree based on the 16S rRNA gene, RNAmmer [73] was first used to predict the 16S rRNA gene in each genome. 16S rRNA gene sequences were then aligned by MAFFT [74] and trimmed by TrimAl [75]. Finally, a maximum likelihood phylogenetic tree was inferred by FastTree v2.1.10 (options–gamma) [66].

### Transcriptomics and quantitative RT-PCR

Cell suspensions of MTRN7 cultured in basal medium with or without glucose were used as two controls, while cell suspensions cultured in the basal medium supplied with arabinan was the experimental group. All cultures were incubated at 28 °C for 5 h in triplicate and frozen in liquid nitrogen immediately. The total RNA was extracted using TRIzol® Reagent, and genomic DNA was then removed via DNase I treatment (TaKaRa). An RNA-seq transcriptome library was then prepared with the TruSeq^TM^ RNA sample preparation Kit (Illumina, San Diego, CA) using 2 μg of a high-quality total RNA. The paired-end RNA-seq library was then sequenced using an Illumina HiSeq×TEN (2 × 150 bp read length) at Shanghai Majorbio Bio-pharm Biotechnology Co., Ltd. (Shanghai, China). After quality control using an in-house perl script, high-quality reads in each sample were mapped to the genome of MTRN7 using Bowtie2 v2.2.3 [76]. Gene expression levels (FPKM, fragments per kilobase of transcript per million mapped reads) were quantified by RSEM [77]. Differentially expressed genes between the control and experimental groups were identified by edgeR [78], with FDR-adjusted *p* < 0.05 and |log_2_FC| > 1 as the cutoff for significantly different expression.

Expression levels of genes of interest were verified by quantitative RT-PCR. Extracted RNA used for transcriptome sequencing was converted into cDNA via the PrimeScript RT Reagent Kit. qRT-PCR was performed on the QuantStudio™ 5 Real-Time PCR System (Applied Biosystems, Foster City, CA, USA) based on the gene-specific primers listed in Table S10. The fold change of gene transcription for each gene was normalized using the housekeeping gene *recA* as a reference. All assays were performed in triplicate.

### Heterologous expression, purification and characterization of Abf51A

The *abf51A* gene was initially amplified with a self-designed primer pair (Table S11) and cloned into pET28 (+). The resulting vectors containing *abf51A* (without signal peptides) were then introduced into *E. coli* BL21 (DE3) for downstream expression and purification. Purified Abf51A was assessed by 12% sulfate-polyacrylamide gel electrophoresis (SDS-PAGE). The enzyme activity of Abf51A on synthetic *p*NP-α-L-arabinofuranose (Sigma) was measured by monitoring the release of *p*NP-OH spectrophotometrically (405 nm) using a microtiter plate absorbance reader. In triplicate, 10 μl enzyme and 10 μl *p*NP-α-L-arabinofuranose (20 mM) were added to 180 μl potassium phosphate buffer (50 mM, pH 6.2) to form a reaction system. To test enzymatic activities under different temperatures, the reactions were operated at 4, 10, 16, 28, 37, 45, 50, 60, and 70 °C. To investigate enzymatic activities under high-pressure and low-temperature conditions, the reaction was also operated under different pressures (0.1, 10, 20, 30, 40, 50, 60, 70, and 80 Mpa) at 2 °C (in situ temperature of hadal zones) and 25 °C (optimal temperature). High pressure was applied by delivering water using syringes as described above. After a 10-min reaction time, 600 μL of cold 1M Na_2_CO_3_ was added to stop the reaction and the absorbance was determined via a microtiter plate absorbance reader. Specific activity was determined by comparing the experimental absorbance values to a *p*NP-OH standard curve ranging from 0 to 1 mM. One unit of activity was defined as the amount of enzyme required to release 1 μmol of *p*NP-OH per min.

### Supplementary Information

**Additional file 1:** **Fig. S1.** The distribution of *Bacteroidetes* in the Mariana Trench water column based on 16S rRNA gene sequencing from Nunoura *et al.* [2]. **Fig. S2.** NMDS (Non-metric Multi-Dimensional Scaling) analysis of *Bacteroidetes* community across all samples. The shaded ellipses represent the 80% confidence interval. **Fig. S3.** NMDS analysis of *Bacteroidetes* GH and PL genes across all samples. The shaded ellipses represent the 80% confidence interval. **Fig. S4.** Relative abundance (percent of mapped reads) of *Bacteroidetes *MAGs in the Mariana Trench, southern North Sea (algae bloom) and surface Pacific Ocean. **Fig. S5.** Bray-Curtis dissimilarities of *Bacteroidetes* MAGs illustrated by NMDS analysis based on the composition of GH and PL genes in each MAG. The shaded ellipses represent the 80% confidence interval. **Fig. S6.** Phylogenetic tree of MTRN7 with closely related taxa based on 16S rRNA gene sequences using *Olleya aquimaris* DSM 24464 as an outgroup. **Fig. S7.** High-pressure endurance of strain MTRN7 at room temperature when supplied with nutrient-rich 2216E medium. **Fig. S8.** Relative abundance of MTRN7 arabinan PUL across the whole water column of the Mariana Trench. **Fig. S9.** Predicted pathway for the degradation of arabinans in strain MTRN7. Locus tags (APDGNPDO_#) are indicated by the numbers given in parentheses. Based on the results of SignalP [3], enzymes with signal peptides were considered as secretory enzymes associated with the periplasmic space and/or to the outer membrane while those without signal peptides were considered as cytoplasmic proteins. Arabinans are firstly degraded into arabino-oligosaccharides and then transferred into the periplasm through the SusC/D transport system. These oligo-arabinoses can be further degraded into lower-level oligo-arabinose or arabinose, which are transported into the cytoplasm by an MFS transporter. In the cytoplasm, all oligo-arabinoses are decomposed into arabinoses. Finally, arabinose isomerase converts arabinose into ribulose, which is then converted to ribulose-5-phosphate which enters the pentose phosphate pathway. XynD (Arabinoxylan arabinofuranohydrolase, APDGNPDO_02643) is not shown in this plot considering its function in cleaving arabinose units from O-2- or O-3-monosubstituted xylose residues from arabinoxylan. SSF (sodium/sugar cotransporter, APDGNPDO_02653) and SusB (alpha-glucosidase, APDGNPDO_02654) are also not included, both of which are likely involved in glucan degradation and absorption. **Fig. S10.** Maximum likelihood trees of CAZymes from GH43, GH51 and GH127 families located in the MTRN7 arabinan PUL, a GTPase (APDGNPDO_02636) located 30 genes upstream of the arabinan PUL and a GH43_28 enzyme (APDGNPDO_01973) outside the arabinan PUL. The tree of each enzyme was constructed with 100 homologues downloaded from NCBI. Enzymes from MTRN7 were marked in red font. These enzymes were grouped based on their isolated environments: Black, terrestrial or freshwater; blue, marine. **Fig. S11.** Growth of MTRN7 under different pressures at 2 °C using marine minimal medium (MMM) supplied with 2% arabinan as the carbon source.

**Additional file 2:** **Table S1.** Metagenomes used in this study. **Table S2.** Relative abundance (copies per million reads) of *Bacteroidetes* CAZyme genes in different samples. **Table S3.** Genome information of MAGs used in this study. **Table S4.** Genome relative abundance (percent of mapped reads) of all MAGs in metagenomic samples. **Table S5.** Predicted PULs in all MAGs used in this study. **Table S6.** Distribution of CAZyme genes in all MAGs. **Table S7. **Genome information of *Mesoflavibacter* genomes used in this study. **Table S8.** Pairwise comparation of genes from the arabinan PULs in *Mangrovimonas* sp. ST2L15 and *Mesoflavibacter* *profundi* MTRN7. **Table S9.** Components of marine mineral medium (MMM). **Table S10. **The primers used for RT-qPCR. **Table S11.**The primers used for gene cloning. The cleavage sites are underlined.

## Data Availability

The whole genome sequence of *M. profundi* MTRN7 was deposited at GenBank under the accession number PRJNA898768. The raw sequencing reads for transcriptomic analysis have been deposited to the NCBI Short Read Archive (accession number: PRJNA898788). Information of metagenomes and MAGs used in this study were listed in Table S1 and Table S3, respectively.
