# Supplementary material for: Deep-sea Bacteroidetes from the Mariana Trench specialize in hemicellulose and pectin degradation typically associated with terrestrial systems
Source: Microbiome. 2023 Aug 7;11:175. doi: 10.1186/s40168-023-01618-7 (PMC10405439; doi:10.1186/s40168-023-01618-7)
Supplement: Supplementary file 2 — Additional file 1: Fig. S1. The distribution of Bacteroidetes in the Mariana Trench water column based on 16S rRNA gene sequencing from Nunoura et al. [2]. Fig. S2. NMDS (Non-metric Multi-Dimensional Scaling) analysis of Bacteroidetes community across all samples. The shaded ellipses represent the 80% confidence interval. Fig. S3. NMDS analysis of Bacteroidetes GH and PL genes across all samples. The shaded ellipses represent the 80% confidence interval. Fig. S4. Relative abundance (percent of mapped reads) of Bacteroidetes MAGs in the Mariana Trench, southern North Sea (algae bloom) and surface Pacific Ocean. Fig. S5. Bray-Curtis dissimilarities of Bacteroidetes MAGs illustrated by NMDS analysis based on the composition of GH and PL genes in each MAG. The shaded ellipses represent the 80% confidence interval. Fig. S6. Phylogenetic tree of MTRN7 with closely related taxa based on 16S rRNA gene sequences using Olleya aquimaris DSM 24464 as an outgroup. Fig. S7. High-pressure endurance of strain MTRN7 at room temperature when supplied with nutrient-rich 2216E medium. Fig. S8. Relative abundance of MTRN7 arabinan PUL across the whole water column of the Mariana Trench. Fig. S9. Predicted pathway for the degradation of arabinans in strain MTRN7. Locus tags (APDGNPDO_#) are indicated by the numbers given in parentheses. Based on the results of SignalP [3], enzymes with signal peptides were considered as secretory enzymes associated with the periplasmic space and/or to the outer membrane while those without signal peptides were considered as cytoplasmic proteins. Arabinans are firstly degraded into arabino-oligosaccharides and then transferred into the periplasm through the SusC/D transport system. These oligo-arabinoses can be further degraded into lower-level oligo-arabinose or arabinose, which are transported into the cytoplasm by an MFS transporter. In the cytoplasm, all oligo-arabinoses are decomposed into arabinoses. Finally, arabinose isomerase converts arabinose i [file 40168_2023_1618_MOESM1_ESM.docx]

**Supplementary materials**

**Supplementary results**

To further validate a role in horizontal gene transfer, we constructed maximum likelihood trees with 100 homologues obtained from NCBI nr database using seven key degradative enzymes from GH43, GH51 and GH127 families in the PUL as query sequences (Fig. S10). For comparison, phylogenetic trees for a GTPase (APDGNPDO_02636) located just three genes upstream of the PUL and a GH43_28 β-xylanase (APDGNPDO_01973) from a distinct genomic region were also generated (Fig. S10). Our results showed many homologues of these genes located in the PUL were from terrestrial *Bacteroidetes*. However, the GTPase and the GH43_28 β-xylanase outside the PUL were almost exclusively populated with marine sequences. Similar phenomenon was also observed in the marine bacteria *Pseudoalteromonas haloplanktis* ANT/505, which possessed a pectin PUL with a potential terrestrial origin [1]. We thus inferred that the arabinan PUL in MTRN7 was most likely acquired from coastal or terrestrial microbes by an ancestor of *Mesoflavibacter profundi*. In view of a highly similar PUL in a mangrove forest originated *Bacteroidetes*, the arabinan PUL might provide competitive advantage for MTRN7 in degrading plant originated arabinan.

**Supplementary figures**

**Fig. S1** The distribution of *Bacteroidetes* in the Mariana Trench water column based on 16S rRNA gene sequencing from Nunoura *et al*. [2].

**
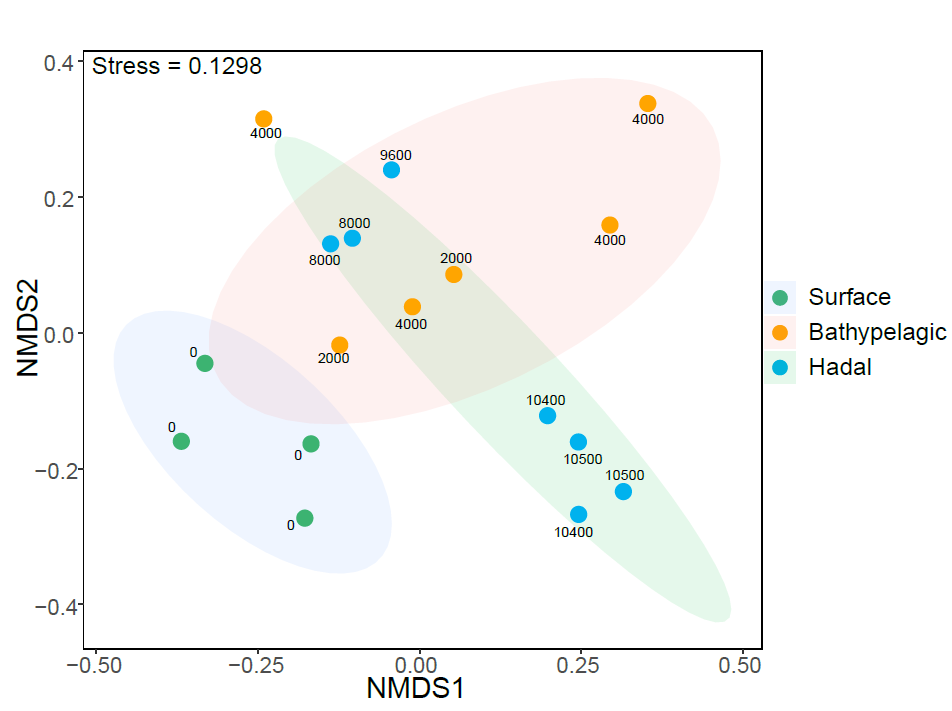
**

**Fig. S2** NMDS (Non-metric Multi-Dimensional Scaling) analysis of *Bacteroidetes* community across all samples. The shaded ellipses represent the 80% confidence interval.


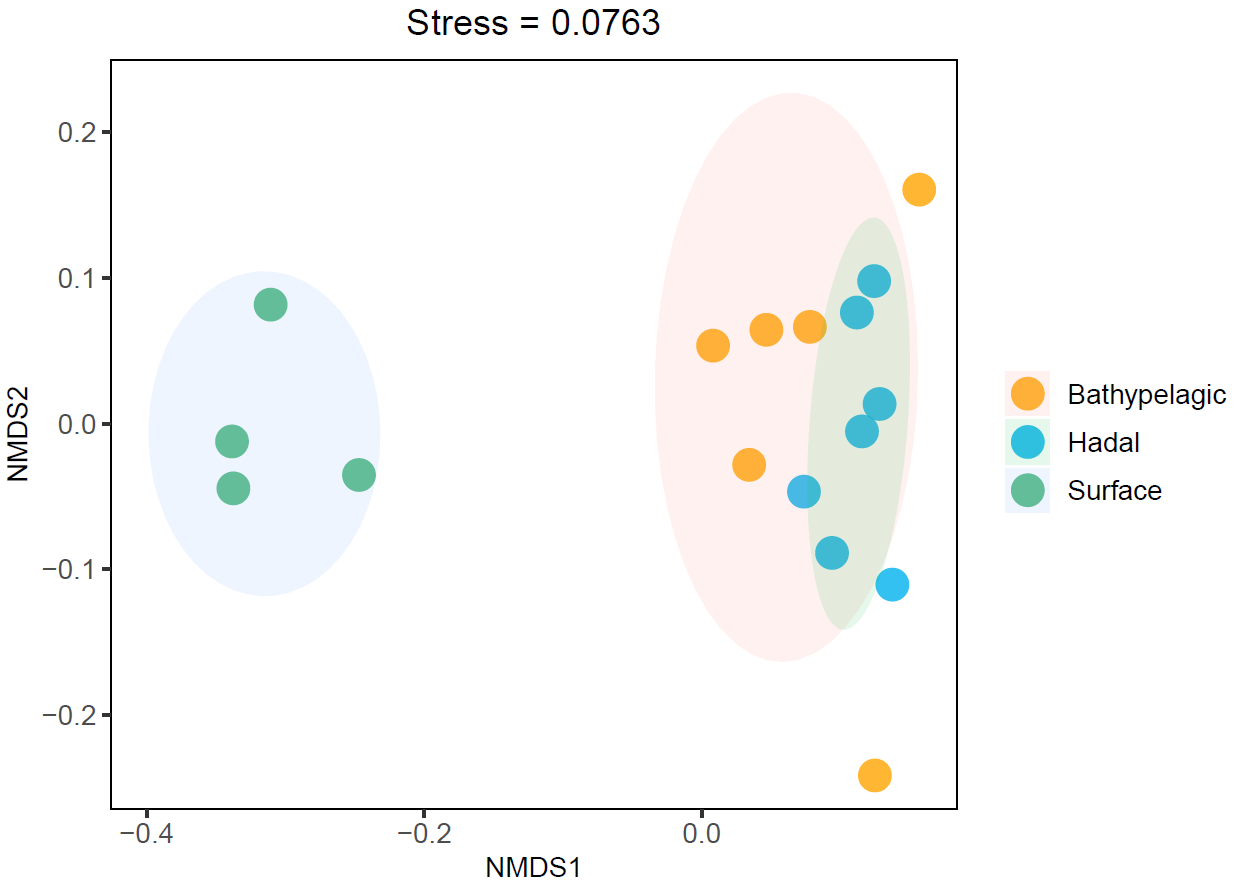


**Fig. S3** NMDS analysis of *Bacteroidetes* GH and PL genes across all samples. The shaded ellipses represent the 80% confidence interval.

**
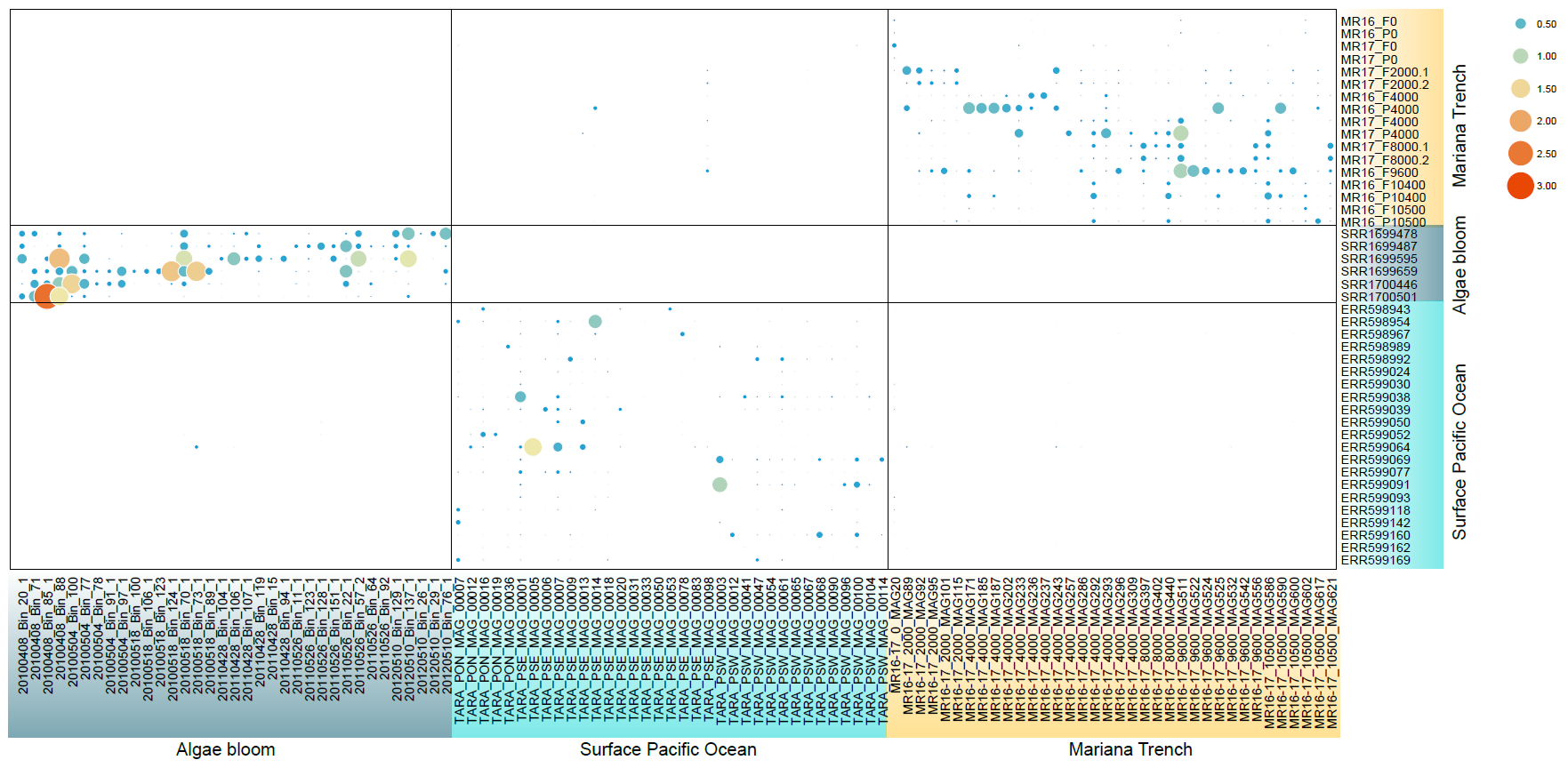
**

**Fig. S4** Relative abundance (percent of mapped reads) of *Bacteroidetes* MAGs in the Mariana Trench, southern North Sea (algae bloom) and surface Pacific Ocean.


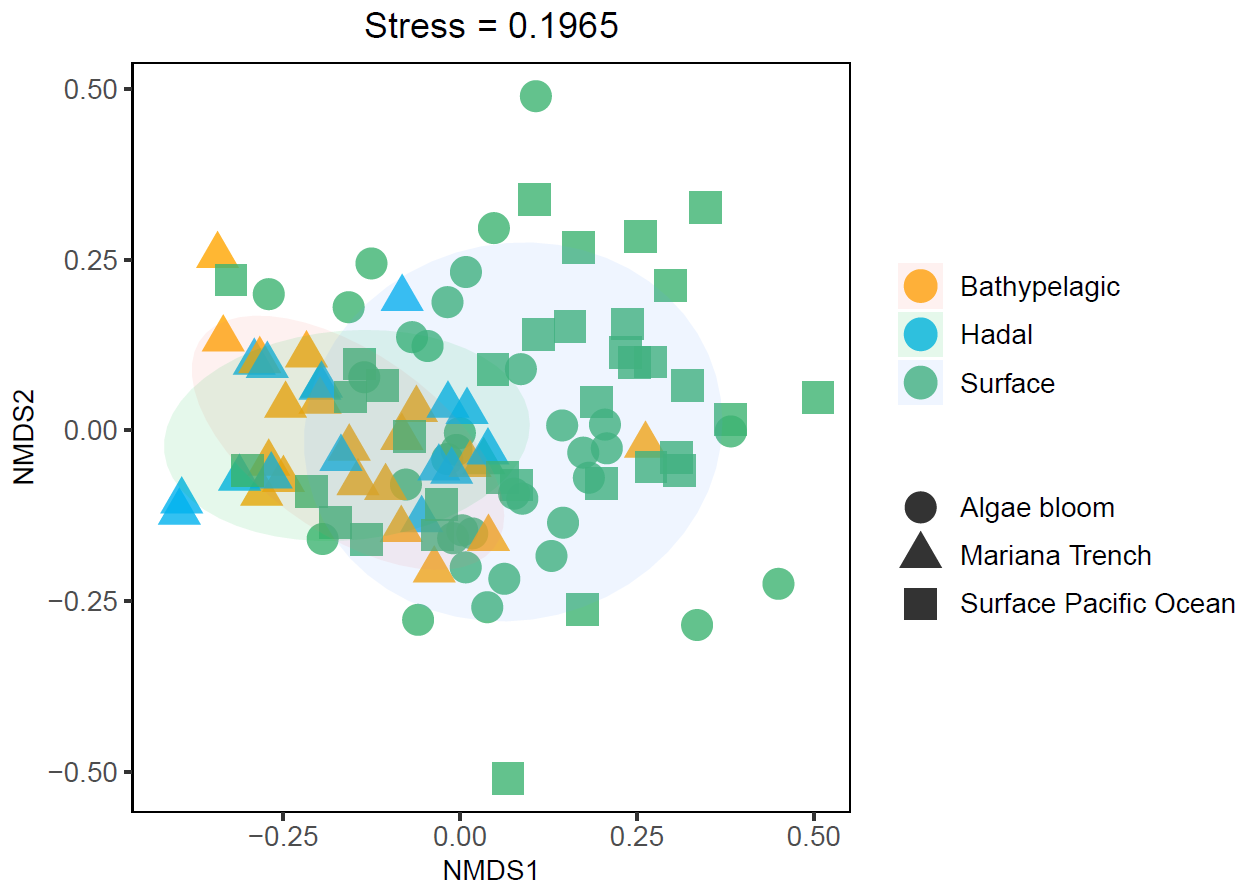


**Fig. S5** Bray-Curtis dissimilarities of *Bacteroidetes* MAGs illustrated by NMDS analysis based on the composition of GH and PL genes in each MAG. The shaded ellipses represent the 80% confidence interval.


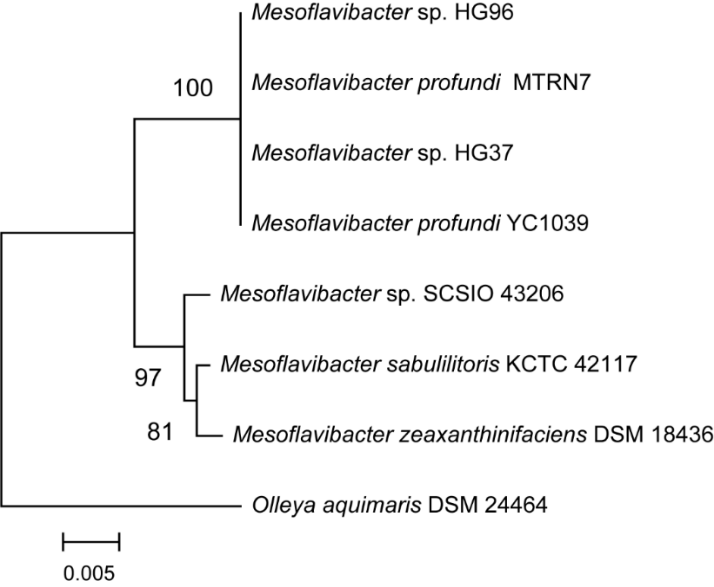


**Fig. S6** Phylogenetic tree of MTRN7 with closely related taxa based on 16S rRNA gene sequences using *Olleya aquimaris* DSM 24464 as an outgroup.

**
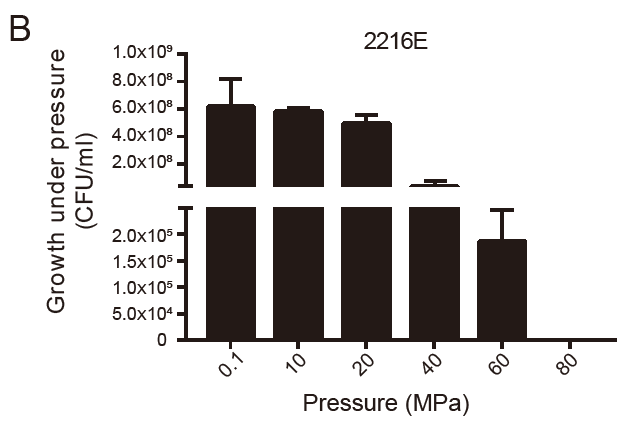
**

**Fig. S7** High-pressure endurance of strain MTRN7 at room temperature when supplied with nutrient-rich 2216E medium.


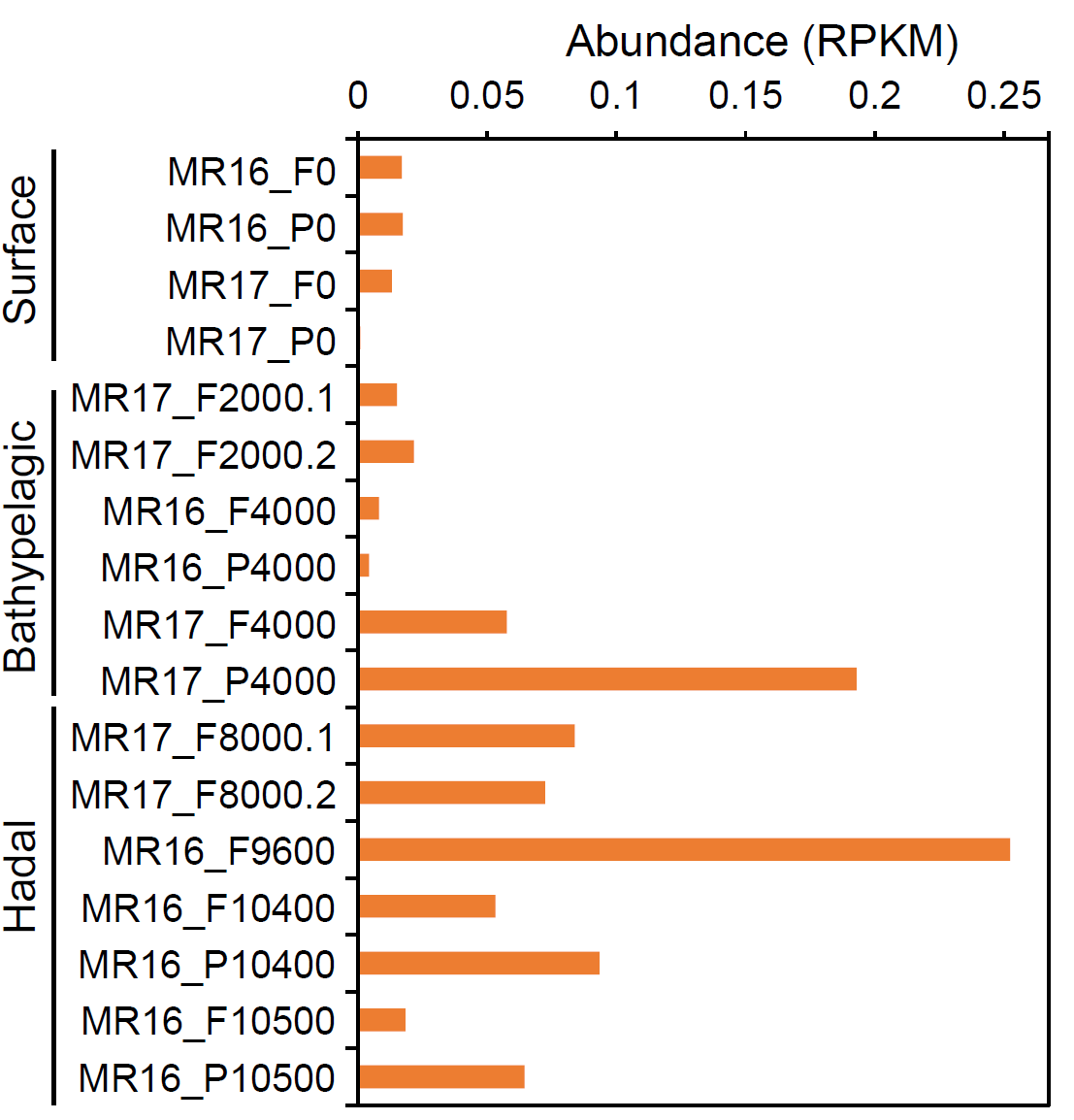


**Fig. S8** Relative abundance of MTRN7 arabinan PUL across the whole water column of the Mariana Trench.

**
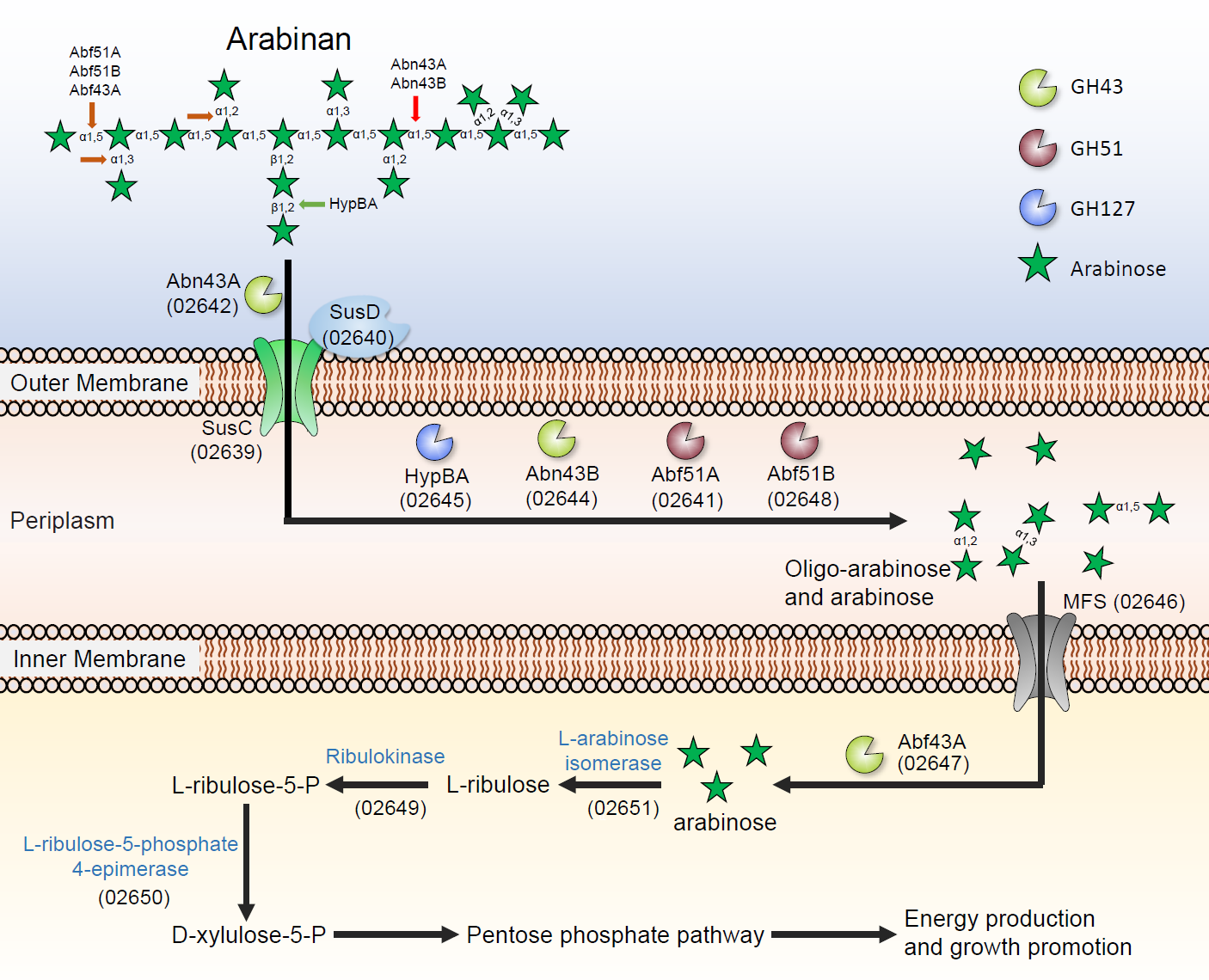
**

**Fig. S9** Predicted pathway for the degradation of arabinans in strain MTRN7. Locus tags (APDGNPDO_#) are indicated by the numbers given in parentheses. Based on the results of SignalP [3], enzymes with signal peptides were considered as secretory enzymes can either be associated with the periplasmic space and/or to the outer membrane while those without signal peptides were considered as cytoplasmic proteins. Arabinans are firstly degraded into arabino-oligosaccharides and then transferred into the periplasm through the SusC/D transport system. These oligo-arabinoses can be further degraded into lower-level oligo-arabinose or arabinose, which are transported into the cytoplasm by an MFS transporter. In the cytoplasm, all oligo-arabinoses are decomposed into arabinoses. Finally, arabinose isomerase converts arabinose into ribulose, which is then converted to ribulose-5-phosphate to enter the pentose phosphate pathway. XynD (Arabinoxylan arabinofuranohydrolase, APDGNPDO_02643) is not shown in this plot considering its function in cleaving arabinose units from O-2- or O-3-monosubstituted xylose residues from arabinoxylan. SSF (sodium/sugar cotransporter, APDGNPDO_02653) and SusB (alpha-glucosidase, APDGNPDO_02654) are also not included, both of which are likely involved in glucan degradation and absorption.


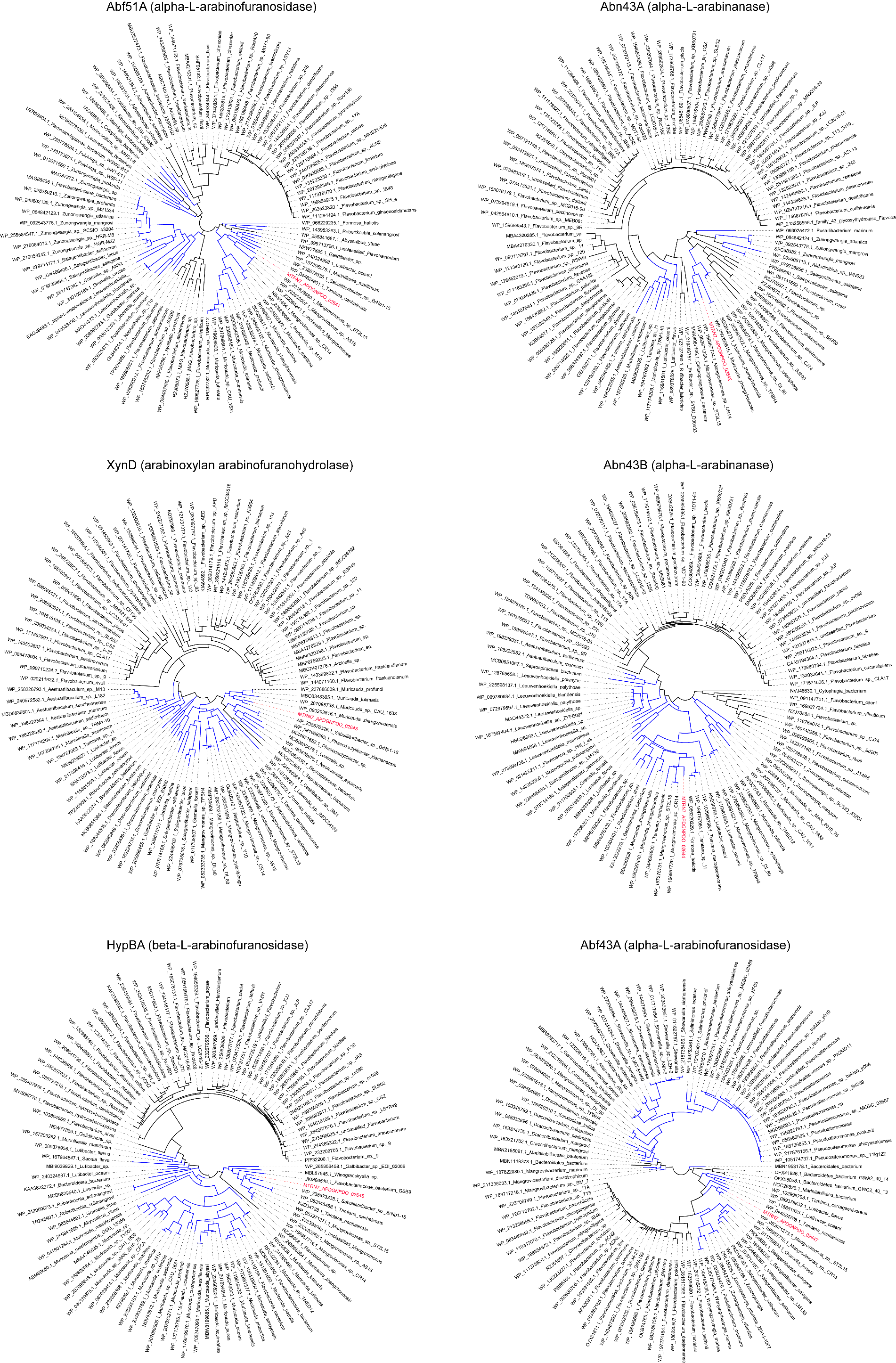


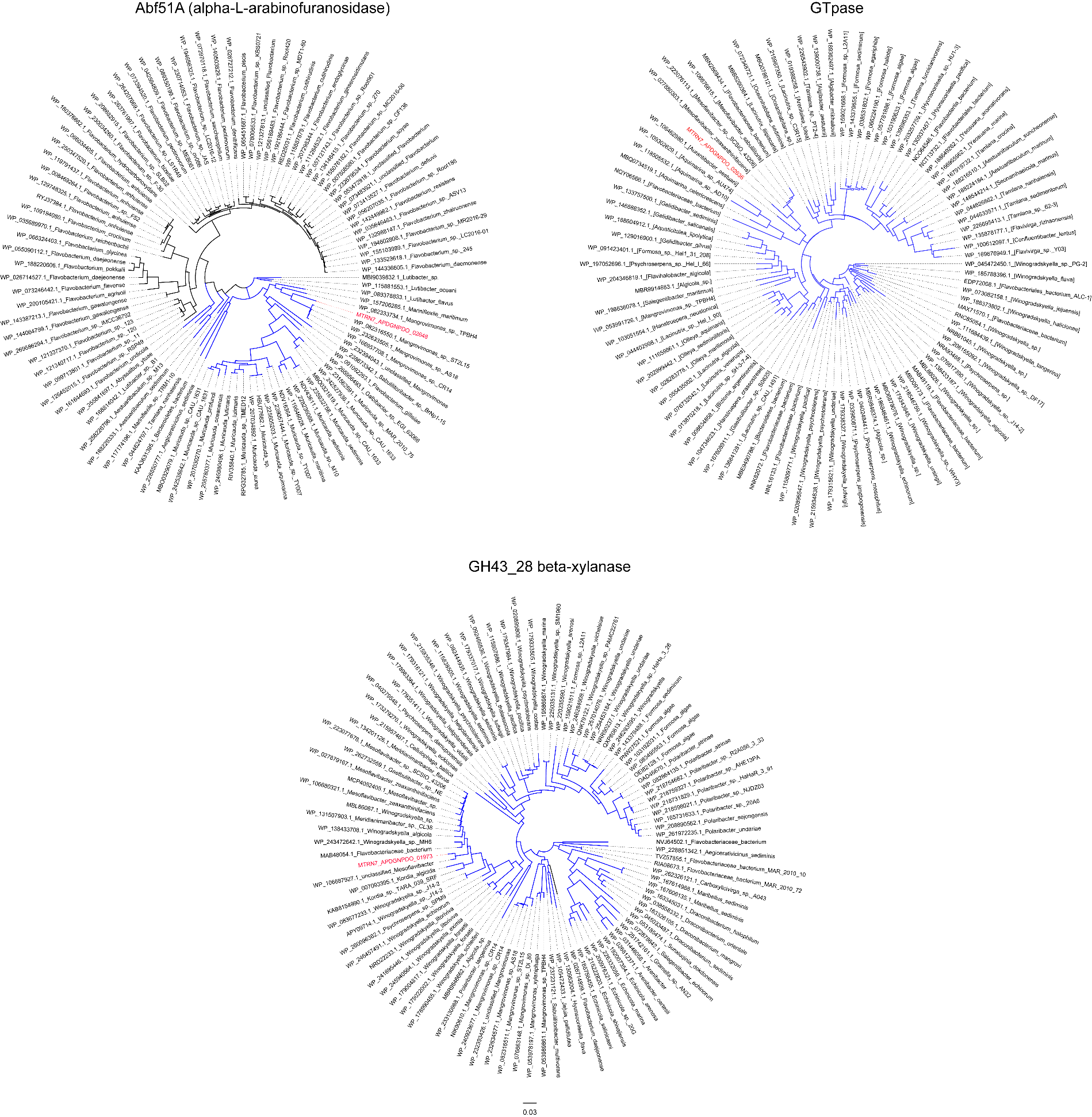


**Fig. S10** Maximum likelihood trees of CAZymes from GH43, GH51 and GH127 families located in the MTRN7 arabinan PUL, a GTPase (APDGNPDO_02636) located 30 genes upstream of the arabinan PUL and a GH43_28 enzyme (APDGNPDO_01973) outside the arabinan PUL. Tree of each enzyme was constructed with 100 homologues downloaded from NCBI. Enzymes from MTRN7 were marked in red font. These enzymes were grouped based on their isolated environments: Black, terrestrial or freshwater; blue, marine.

**Fig. S11** Growth of MTRN7 under different pressures at 2 ℃ using marine minimal medium (MMM) supplied with 2% arabinan as the carbon source.

**References:**

1. Hehemann JH, Truong LV, Unfried F, Welsch N, Kabisch J, Heiden SE, et al. Aquatic adaptation of a laterally acquired pectin degradation pathway in marine gammaproteobacteria. Environ Microbiol. 2017;19:2320-33.

2. Nunoura T, Takaki Y, Hirai M, Shimamura S, Makabe A, Koide O, et al. Hadal biosphere: insight into the microbial ecosystem in the deepest ocean on Earth. Proc Natl Acad Sci U S A. 2015;112:E1230-36.

3. Teufel F, Almagro Armenteros JJ, Johansen AR, Gislason MH, Pihl SI, Tsirigos KD, et al. SignalP 6.0 predicts all five types of signal peptides using protein language models. Nat Biotechnol. 2022;40:1023-25.
